# A mechanism of self-lipid endocytosis mediated by the receptor Mincle

**DOI:** 10.1073/pnas.2120489119

**Published:** 2022-07-22

**Authors:** Alexey V. Kostarnoy, Petya G. Gancheva, Igor I. Kireev, Andrey I. Soloviev, Bernd Lepenies, Andrey Y. Kulibin, Ekaterina A. Malolina, Olga N. Scheglovitova, Alexey V. Kondratev, Maria V. Sokolova, Daria Vorobyeva, Nadezhda Filippova, Marina R. Kapkaeva, Maria A. Lagarkova, Pavel S. Metalnikov, Evgeniy Riabenko, Daniil A. Grumov, Maxim A. Bobrov, Elena Vasilieva, Boris S. Naroditsky, Alexander L. Gintsburg

**Affiliations:** ^a^Laboratory of Rickettsial Ecology, N. F. Gamaleya National Research Center of Epidemiology and Microbiology, 123098 Moscow, Russia;; ^b^Laboratory of Immunobiotechnology, N. F. Gamaleya National Research Center of Epidemiology and Microbiology, 123098 Moscow, Russia;; ^c^Belozersky Institute of Physico-Chemical Biology, Lomonosov Moscow State University, 119991 Moscow, Russia;; ^d^Laboratory of Genetic Mechanisms of Development, V.I. Kulakov National Medical Research Center for Obstetrics, Gynecology, and Perinatology, 117997 Moscow, Russia;; ^e^Laboratory of Detection and Ultrastructural Analysis of Microorganisms, N. F. Gamaleya Federal Research Center of Epidemiology and Microbiology, 123098 Moscow, Russia;; ^f^Immunology Unit, Research Center for Emerging Infections and Zoonoses, University of Veterinary Medicine, 30559 Hannover, Germany;; ^g^Laboratory of Evolutionary Developmental Biology, Koltzov Institute of Developmental Biology, Russian Academy of Sciences, 119071 Moscow, Russia;; ^h^Laboratory of Antiviral Immunity, N. F. Gamaleya Federal Research Center of Epidemiology and Microbiology, 123098 Moscow, Russia;; ^i^Laboratory of Mediators and Effectors of Immunity, N. F. Gamaleya National Research Center of Epidemiology and Microbiology, 123098 Moscow, Russia;; ^j^Laboratory of Atherothrombosis, Moscow State University of Medicine and Dentistry, 127473 Moscow, Russia;; ^k^Federal Research and Clinical Center of Physical-Chemical Medicine, Federal Medical-Biological Agency, 119435 Moscow, Russia;; ^l^Department of Pathology, M. F. Vladimirsky Moscow Regional Clinical Research Institute, 129110 Moscow, Russia;; ^m^Laboratory of Gene Engineering of Pathogenic Microorganisms, N. F. Gamaleya National Research Center of Epidemiology and Microbiology, 123098 Moscow, Russia

**Keywords:** endocytosis, C-type lectin receptors, Mincle, endothelial cells, ganglioside

## Abstract

Cellular lipid uptake (through endocytosis) is a basic physiological process. Dysregulation of this process underlies the pathogenesis of diseases such as atherosclerosis, obesity, diabetes, and cancer. However, to date, only some mechanisms of lipid endocytosis have been discovered. Here, we show a previously unknown mechanism of lipid cargo uptake into cells mediated by the receptor Mincle. We found that the receptor Mincle, previously shown to be a pattern recognition receptor of the innate immune system, tightly binds a range of self-lipids. Moreover, we revealed the minimal molecular motif in lipids that is sufficient for Mincle recognition. Superresolution microscopy showed that Mincle forms vesicles in cytoplasm and colocalizes with added fluorescent lipids in endothelial cells but does not colocalize with either clathrin or caveolin-1, and the added lipids were predominantly incorporated in vesicles that expressed Mincle. Using a model of ganglioside GM3 uptake in brain vessel endothelial cells, we show that the knockout of Mincle led to a dramatic decrease in lipid endocytosis. Taken together, our results have revealed a fundamental lipid endocytosis pathway, which we call Mincle-mediated endocytosis (MiME), and indicate a prospective target for the treatment of disorders of lipid metabolism, which are rapidly increasing in prevalence.

## Significance

Dysregulation of lipid endocytosis, a normal physiological process of cellular lipid uptake, often underlies the pathogenesis of some widespread diseases such as atherosclerosis, obesity, and diabetes. However, the mechanisms of lipid endocytosis are incompletely understood, and only a few such mechanisms have been discovered, limiting the available therapeutic strategies and targets in these diseases. Here we found that the receptor Mincle, previously known as a pattern recognition receptor of the innate immune system, plays a significant role in endocytosis. The results have revealed a fundamental pathway of lipid endocytosis, which we call Mincle-mediated endocytosis.

Some endocytic pathways in eukaryotic cells have been described, and the major endocytic routes for the internalization of many cargoes are clathrin- and caveolae-mediated endocytosis (1–3). Additionally, some C-type lectins are important players in endocytic processes, including lectin-like oxidized low-density lipoprotein receptor-1, which drives the endocytosis of acetylated low-density lipoproteins (4); thrombomodulin, which drives thrombin internalization (5); and galectin-3, which drives the formation of clathrin-independent carriers for cargo internalization (6). Furthermore, the glycolipid–lectin hypothesis, which explains how cell wall glycolipids and lectins mediate nanoenvironments from which glycosylated cargo proteins endocytose, was recently proposed (1). One of the C-type lectin receptor, Mincle (Clec4e), is considered a pattern recognition receptor of the innate immune system which recognizes both self-lipids and non-self-lipids (7, 8). Early studies showed that Mincle recognizes bacterial and fungal glycolipids (9, 10). Additionally, Mincle recognizes endogenous cholesterol (11) and cholesterol sulfate (12), inducing an inflammatory response. Recently, one of the structurally simplest glycosphingolipids (GSLs), β-glucosylceramides, was shown to bind Mincle on myeloid cells and induce immediate inflammatory responses (13). However, to date Mincle’s role in lipid uptake is unclear. As Mincle is a pattern recognition receptor, we hypothesized that it may recognize a broad range of lipids with similar structure and may play role in lipid endocytosis.

## Results

### Endothelial Cells Express Mincle.

**Fig. 1.**
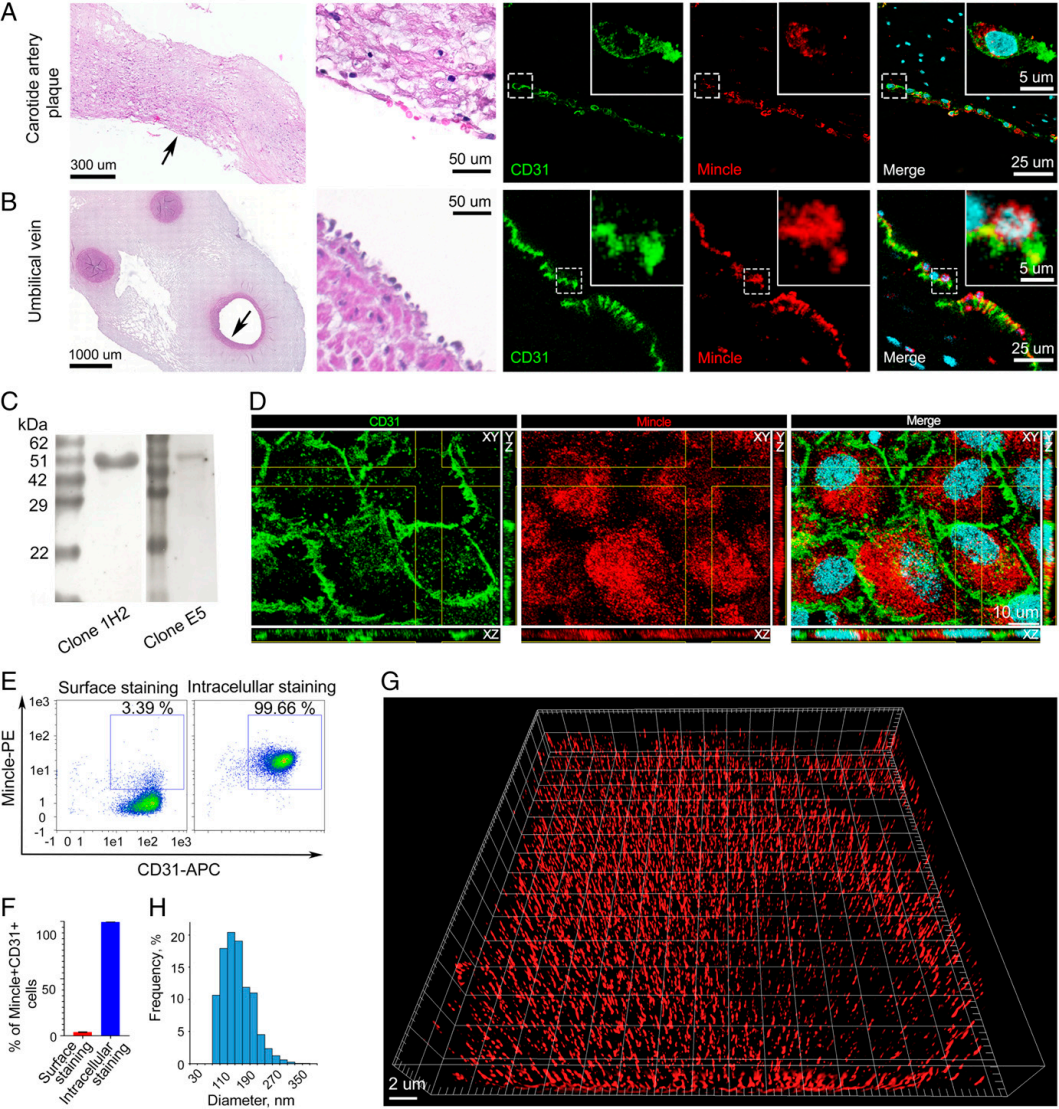
Human endothelial cells express Mincle. (*A*) Hematoxylin/eosin and immunohistochemical staining using antibodies against Mincle (red) and CD31 (green) in human carotid plaque specimens collected immediately after endarterectomy. Nuclei were costained with DAPI (cyan); *n* = 6 samples. Representative images are shown. (*B*) Hematoxylin/eosin and immunohistochemical staining of human umbilical cord specimens for Mincle (red) and CD31 (green). Nuclei were costained with DAPI (cyan); *n* = 6 samples. Representative images are shown. (*C*) HUVEC lysates were evaluated for Mincle expression by Western blotting using two clones of monoclonal antibodies. (*D*) HUVECs were costained for Mincle (red) and CD31 (green) and imaged by confocal microscopy. (*E* and *F*) Flow cytometry was performed on HUVECs stained for surface and intracellular Mincle. Flow cytometry plots (*E*) and quantification of Mincle+CD31+ cell percentages (*F*) are depicted. (*G*) SIM of HUVECs stained for Mincle revealed the presence of this receptor in the cytoplasm in small, uniformly sized bodies with an average diameter of 115.2 ± 33.2 nm (mean ± SD); the histogram is presented in *H*; in total, 14,768 Mincle-expressing bodies were measured in *n* = 5 cells with an Imaris 7.2. The results are presented as the mean ± SD values.

GSLs are present in the blood as constituents of lipoproteins (14) and extracellular vesicles (15) and are also found on the outer layer of enveloped viruses (16). Furthermore, large amounts of GSLs transported through endothelium accumulate in fatty streaks and atherosclerotic plaques (17, 18). Because uptake of lipids by endothelium is an important physiologic process we examined Mincle’s role in lipid endocytosis using endothelial cells. First of all, we determined whether Mincle is expressed on endothelium. Immunohistochemical studies of human carotid plaque specimens collected after endarterectomy revealed that Mincle is expressed not only in arterial endothelial cells (Fig. 1*A*, formalin-fixed paraffin-embedded tissue, and *SI Appendix*, Fig. S1*A*, frozen tissue) but also in the endothelium of the vasa vasorum (*SI Appendix*, Fig. S1*B*), a network of blood microvessels that supply the walls of large blood vessels with nutrients and oxygen. Further, we show that not only the endothelium of carotid artery expresses Mincle. The human umbilical vein endothelium in umbilical cords was found to also express Mincle, according to immunohistochemical evaluation (Fig. 1*B*). As human umbilical vein endothelial cells (HUVECs) constitute a well-established model system in which many aspects of endothelial function and disease, including endocytosis, are studied, we used HUVECs to comprehensively study expression of the receptor Mincle. Sodium dodecyl sulfate polyacrylamide gel electrophoresis under reducing conditions and subsequent Western blotting with two different anti-Mincle antibody clones confirmed the presence of Mincle in the HUVECs (Fig. 1*C*). In contrast to myeloid cells, in which Mincle is usually detected as a monomer (19, 20), in the lysate of HUVECs Mincle was detected in a dimeric form, with a molecular mass of ∼50 kDa that may imply a covalent bond holding the Mincle monomers in endothelial cells. To further verify the presence of the receptor Mincle in the endothelial cells, mass spectrometry was performed. Proteins in HUVEC lysates were separated on a polyacrylamide gel under reducing conditions, after which gel bands corresponding to proteins with a molecular mass of ∼50 kDa were excised and digested with trypsin. The tryptic peptides were then analyzed by liquid chromatography–mass spectrometry (*SI Appendix*, Fig. S2*A*). Mincle was identified by the presence of four peptides (*SI Appendix*, Fig. S2*B*).

After verifying Mincle expression in endothelial cells we next assessed its location in the cell. Confocal immunofluorescence microscopy revealed that Mincle expression was predominantly intracellular (Fig. 1*D*). The predominant intracellular localization of the receptor was also shown via flow cytometry analysis of cells stained for both surface and intracellular Mincle (Fig. 1*E* and *F*). Furthermore, structured illumination superresolution microscopy (SIM) of the HUVECs revealed that Mincle was present in the cytoplasm in small, uniformly sized bodies (vesicles) with an average diameter of 115.2 ± 33.2 nm (Fig. 1 *G* and *H*). The determined average diameter is similar to the diameter of typical clathrin-coated vesicles (∼90 nm) (21). However, SIM revealed that Mincle did not colocalize with either clathrin or caveolin-1 in HUVECs (*SI Appendix*, Figs. S3 and S4).

To study subcellular Mincle localization we stained HUVECs with antibodies specific to Mincle and to some markers of subcellular structures. Confocal immunofluorescence microscopy and SIM revealed Mincle’s presence in the Golgi complex and in membranes of lysosomes (*SI Appendix*, Fig. S5).

### Mincle Recognizes Various GSLs through Direct Binding.

**Fig. 2.**
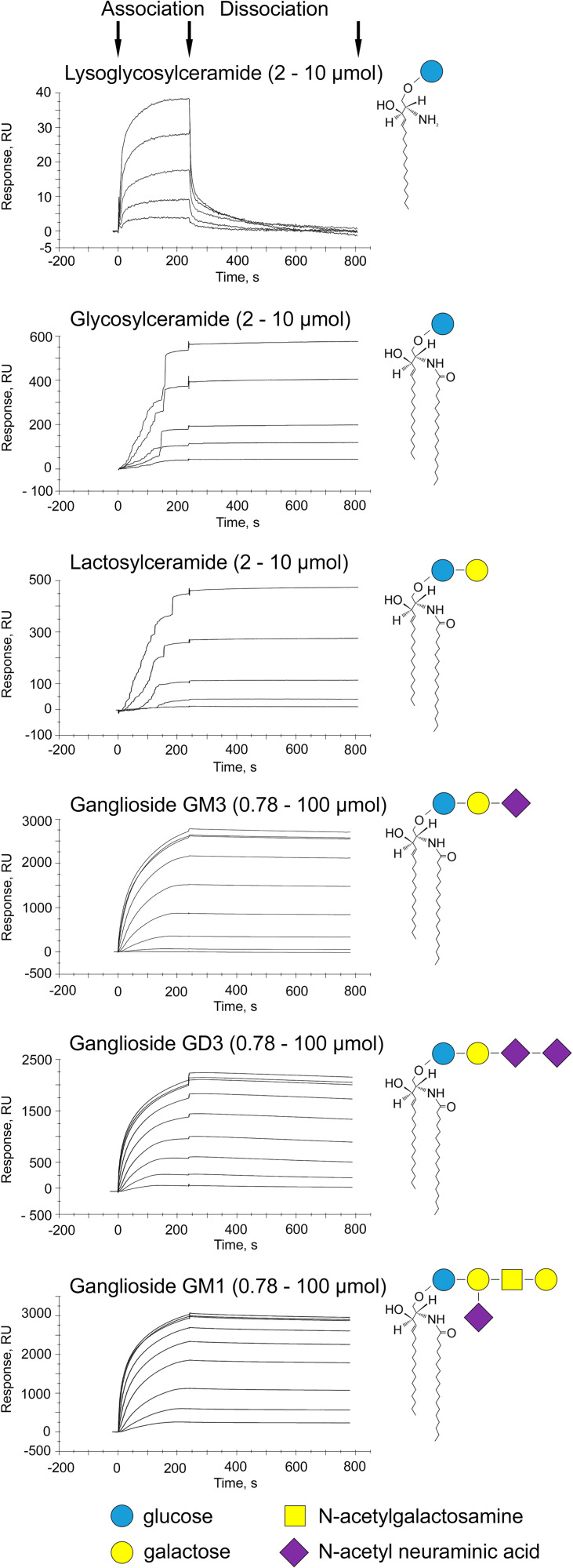
Mincle recognizes various GSLs through direct binding. SPR sensorgrams of glycolipids binding to chip-immobilized Mincle are expressed in response units (RU) vs. time after double referencing (blank surface and blank buffer referencing). Recombinant human Mincle was produced in human cells. The concentrations of lysoglucosylceramide, glucosylceramide, and lactosylceramide were 2, 4, 6, 8, and 10 µM (from bottom to top), and the concentrations of gangliosides GM3, GD3, and GM1 were 0.78, 1.56, 3.13, 6.25, 12.5, 25, 50, 75, and 100 µM (from bottom to top). All data shown are representative of at least two independent experiments.

As Mincle is a pattern recognition receptor and glucosylceramides were recently shown to be ligands of Mincle (13), we hypothesized the presence of a class of lipids that contain a molecular motif recognized by Mincle. To define such a motif, we performed surface plasmon resonance (SPR) experiments to assess the binding of Mincle to a series of glycolipids. Commercially available recombinant human Mincle produced in human cells was covalently immobilized onto the sensor chip surface, and highly purified glycolipids were then injected. We observed the direct binding of Mincle to lysoglucosylceramide, glucosylceramide, lactosylceramide, the gangliosides GM3 and GD3, and the branched glycosylated ganglioside GM1 (Fig. 2). These data show that among the tested lipids the minimal molecular motif recognized by Mincle consists of one glucose residue and one acyl chain, constituting a lysoglucosylceramide moiety. However, lysoglucosylceramide dissociated from Mincle very rapidly, in contrast to lipids with two acyl chains, which dissociated from Mincle very slowly. Variations in the glycosylation of the investigated lipids did not dramatically affect either their ability to bind Mincle or the kinetics of their dissociation from Mincle. Thus, these experiments have shown that Mincle can directly bind GSLs and revealed a molecular motif recognized by Mincle. Notably, this molecular motif is present in most eukaryotic GSLs (22).

### Mincle Colocalizes with Exogenous Fluorescent GSLs in Endothelial Cells.

**Fig. 3.**
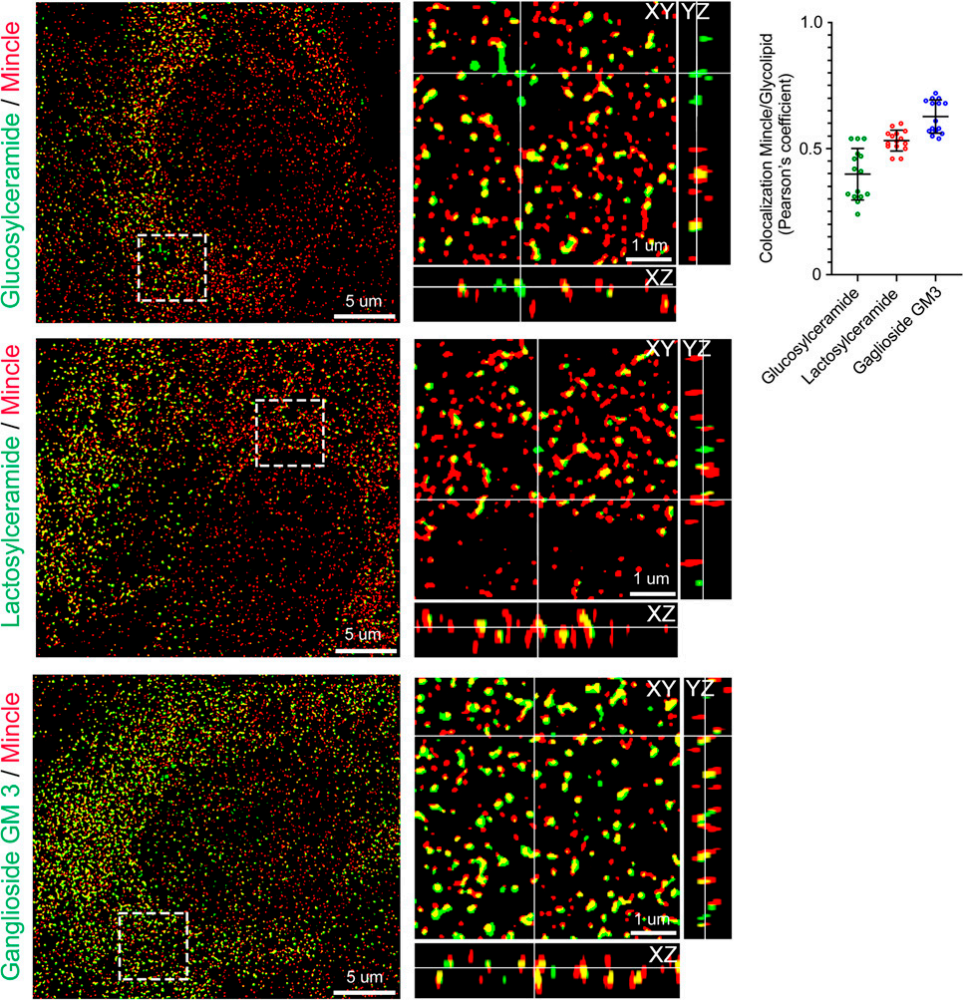
Mincle colocalizes with added fluorescent GSLs in human endothelial cells. (*Left*) Three-dimensional SIM in HUVECs. HUVECs were incubated for 1 h with exogenously added fluorescent GSLs, washed, fixed, labeled with antibodies against Mincle, and analyzed by SIM. Glucosylceramide and lactosylceramide fluorescently labeled with NBD and ganglioside GM3 fluorescently labeled with TopFluor were used. Fluorescent glycoshingolipids are pseudocolored in green and Mincle in red; colocalization is indicated by yellow pseudocoloring. Representative cells are shown. Two independent experiments were performed. (*Right*) Statistical analyses of GSL/Mincle colocalization using the Pearson correlation coefficient; *n* = 15 independent fields per quantification. The results are presented as the mean ± SD values.

SPR experiments revealed that Mincle directly binds a wide range of lipids. To determine whether Mincle could colocalize with added lipids in vitro, we performed experiments using a panel of fluorescently labeled GSLs. In these experiments, we used glucosylceramide and lactosylceramide labeled with the fluorescence molecule 4-nitrobenzo-2-oxa-1, 3-diazole (NBD) and the ganglioside GM3 labeled with the fluorescent dye TopFluor. HUVECs were incubated for 1 h with the added fluorescent lipids, washed, fixed, labeled with antibodies against Mincle, and analyzed by SIM (Fig. 3). Colocalization analysis based on the Pearson correlation coefficient revealed that Mincle colocalized with fluorescent glucosylceramide, lactosylceramide, and GM3, and among the tested glycolipids GM3 had the highest levels of colocolization with the receptor Mincle (Fig. 3, *Right*). Three-dimensional superresolution microscopy revealed that after 1 h of incubation the fluorescent GSLs were predominantly incorporated in vesicles that expressed Mincle (Fig. 3), and the observations suggest the transfer of the lipid cargo via the Mincle-expressing vesicles.

### Mincle Mediates Lipid Endocytosis.

As Mincle was found to colocalize with added fluorescent GSLs, we hypothesized that Mincle plays an important role in the process of lipid endocytosis. We used a cell model of ganglioside GM3 uptake by brain vessel endothelial cells isolated from Mincle-knockout (Mincle-KO) and wild-type (WT) mice to comprehensively evaluate the role of Mincle in the process of lipid endocytosis. As the method of cells’ isolation can affect their functions, we used two different cell isolation protocols. After digestion and preparation of a single-cell suspension, we used either magnetic-activated cell sorting (MACS) (*SI Appendix*, Fig. S6*A*) or puromycin selection (*SI Appendix*, Fig. S7*A*) to isolate brain endothelial cells. Mincle expression and localization were studied in endothelial cells isolated from mouse brain via Western blotting and flow cytometry, confocal microscopy, and SIM (*SI Appendix*, Figs. S6 and S7). Collectively, the data showed that Mincle is expressed in mouse brain endothelial cells, and the receptor Mincle is predominantly intracellular. These observations were consistent between cells isolated using MACS or puromycin selection (*SI Appendix*, Figs. S6 and S7). Notably, the Mincle expression pattern in these cells is similar to that in HUVECs.

**Fig. 4.**
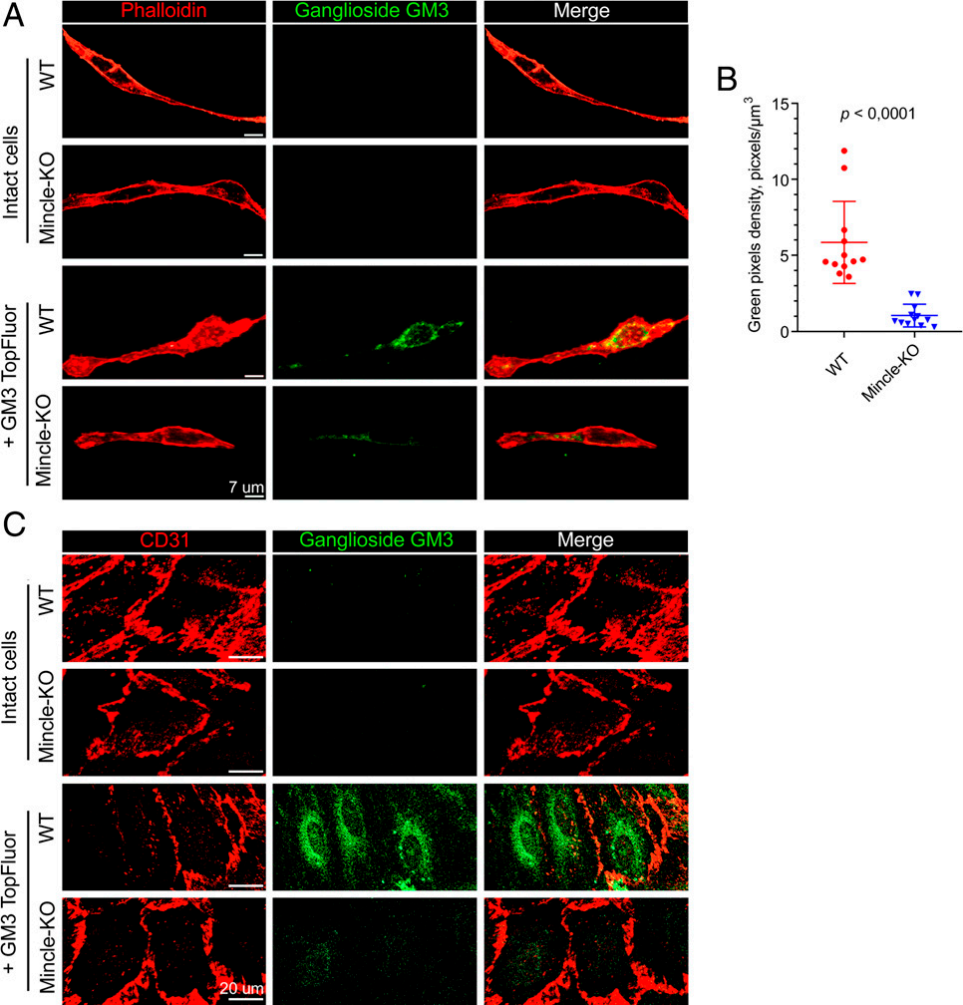
Mincle is necessary for rapid uptake of ganglioside GM3 by brain endothelial cells. (*A*) Brain endothelial cells isolated from Mincle-deficient mice and WT mice using MACS were incubated with fluorescent TopFluor-labeled ganglioside GM3 for 2 h, stained with phalloidin to delineate their boundaries, and evaluated by confocal microscopy. Phalloidin is pseudocolored in red and GM3 in green. (*B*) Quantification of fluorescent GM3 uptake by endothelial cells isolated from Mincle-deficient mice and WT mice using MACS by confocal microscopy. (*C*) Brain endothelial cells isolated from Mincle-deficient (Mincle-KO) mice and WT mice via puromycin selection were incubated with TopFluor-labeled ganglioside GM3 for 2 h, stained using anti-CD31 antibodies to delineate cell boundaries, and evaluated by confocal microscopy. CD31 is pseudocolored in red and GM3 in green. Statistics were calculated with an unpaired two-tailed Student’s *t* test *P < 0.0001. The results are presented as the mean ± SD values.

To evaluate Mincle’s role in the process of lipid endocytosis, ganglioside GM3 labeled with the fluorescent dye TopFluor was added to cultured endothelial cells isolated from the brains of Mincle-KO or WT mice using MACS. After 2 h of incubation, the cells were fixed, stained with phalloidin to delineate their boundaries, and then evaluated by confocal microscopy (Fig. 4*A*). To calculate the quantity of absorbed fluorescent GM3, the pixels inside the cell boundaries were counted (Fig. 4*B*). The data indicated that Mincle KO decreased endocytosis of the ganglioside GM3 by approximately sixfold. A similar evaluation was carried out using a monolayer of mouse endothelial brain cells isolated by puromycin selection. After the addition of fluorescent TopFluor-labeled ganglioside GM3 and incubation for 2 h, the cells were fixed, stained for CD31 to delineate their boundaries, and evaluated via confocal microscopy (Fig. 4*C*). Consistent with the previous experiment with cells isolated by MACS, the results showed that Mincle KO significantly decreased endocytosis of the ganglioside GM3, suggesting that Mincle plays a major role in the uptake of GM3.

## Discussion

Two main routes for the internalization of many cargoes, clathrin- and caveolae-mediated endocytosis, have been intensively studied (1–3). Here, we show a mechanism of self-lipid endocytosis mediated by the receptor Mincle. The newly identified molecular motif recognized by Mincle was found in a range of eukaryotic glycolipids whose structures are conserved from fungi to mammals (22). Interestingly, phylogenetic analysis of the amino acid sequences of Mincle proteins revealed their conservation across different species (23), which suggests that this receptor has important conserved functions. The dual roles of Mincle as an inducer of inflammation in innate immunity and endocytic self-lipids receptor highlight the fundamental parallels between innate immunity and endocytosis (24), and it may be hypothesized that the innate immune system has its evolutionary origin in homeostatic endocytosis processes.

Although Mincle does not contain a clear endocytic motif, it was early shown that pH lowering leads to dramatic reduced Mincle/ligand binding, and the pH profile is very similar to that seen for other endocytic glycan-binding receptors (23). In ref. 25 the authors conclude that Mincle can form covalent heterodimers with MCL, which is an endocytic receptor (26). MCL has a structure similar to that of Mincle and likely arose from gene duplication of Mincle (27). So, coupling with MCL, Mincle can require endocytic receptor functions that allows Mincle in myeloid cells to “signal while eating” (28). It may be hypothesized that MCL can form a covalent complex with Mincle in endothelial cells, too.

The intracellular domain of Mincle in myeloid cells associates with the Fc receptor gamma (FcRγ) signaling chain, which contains an immunoreceptor tyrosine-based activation motif (ITAM), and the ligation of ITAM leads to a signaling cascade that begins with phosphorylation of ITAM tyrosine residues by Src-family kinases, followed by the recruitment and activation of Syk (29, 30). Syk then activates a signaling cascade through CARD9, and this event leads to the induction of inflammatory mediator secretion (30). It can be hypothesized that FcRγ stabilizes Mincle within the lipid bilayer and therefore is involved in the process of lipid endocytosis. To test the hypothesis, ganglioside GM3 labeled with the fluorescent dye TopFluor was added to cultured endothelial cells isolated using puromycin selection from the brains of FcRγ-KO and WT mice. After incubation for 2 h, the cells were fixed, stained for CD31 to delineate their boundaries, and evaluated via confocal microscopy (*SI Appendix*, Fig. S8). As we observed reduced GM3 endocytosis in brain endothelial cells of FcRγ-KO mice, it may be hypothesized that FcRγ assist Mincle in its endocytic function.

Although Mincle in the endothelial cells is predominantly intracellularly located, a smaller portion of the receptor is expressed on cell membrane, and the membrane-located Mincle may be responsible for the lipid uptake. Using specific staining for LAMP1, we have determined Mincle’s presence in the membrane of lysosome. So, it may be hypothesized that Mincle transports endocytosed lipids to utilization in lysosome. Gaucher disease is one of the lysosomal storage diseases in which glucosylceramides accumulate in cells; the disorder is caused by deficiency of the β-GlcCer–degrading enzyme and characterized mainly by systemic inflammation. Recently it was shown that in a Gaucher model in which mice were deficient in the β-GlcCer–degrading enzyme, further deletion of the Mincle gene attenuated inflammatory responses (13). These published observations can be explained as a result of the endocytic role of Mincle, which provides a route for lipid uptake and subsequent transport to lysosome. We have also observed the presence Mincle in Golgi complex. The *trans*-Golgi network (TGN) is the last sorting station of the secretory pathway from which a number of proteins and lipids are sorted for subsequent transport to endocytic compartments (31). So, it is possible that in the TGN the Mincle-containing endosomes are assembled. On the other hand, the presence of Mincle in Golgi can be explained as a result of endosome-to-TGN retrograde traffic.

As GSLs—particularly the ganglioside GM3—are constituents of lipoproteins that circulate in the blood, the endocytic role of Mincle revealed herein may explain the recent report that compared with WT animals Mincle-KO animals showed an altered lipid distribution, reduced hepatic lipid accumulation, and improved glucose metabolism (32). Moreover, Mincle was recently reported to recognize enveloped viruses (33, 34), indicating its possible role in the process of virus entry into cells. As the ganglioside GM3 is a component of the viral envelope that plays a key role in the cellular entry of some viruses (e.g., HIV) (35), the role of Mincle in the endocytosis of enveloped viruses is another issue to be investigated. Unlike in mice, where GM3 exists in more than one form (with either Neu5Gc or Neu5Ac sialic acid), humans only have Neu5Ac GM3, but Neu5Gc GM3 has been reported as a tumor neoantigen (36). In an additional series of experiments, we evaluated whether Mincle can recognize Neu5Gc GM3. According to data obtained via SPR, Neu5Gc GM3 directly binds to Mincle (*SI Appendix*, Fig. S9), and the detailed study of the putative Mincle role in recognizing of Neu5Gc GM3 during carcinogenesis seems to be a promising issue. In addition, our observations may lead to the development of new strategies for receptor-mediated delivery of different forms of drugs. Thus, our results have revealed a fundamental mechanism of lipid endocytosis, which we call MiME, and identified a prospective target for the treatment of diseases that involve disordered lipid metabolism.

## Materials and Methods

C57BL/6, C57BL/6-Mincle-KO, and FcRγ (Fcer1g)-KO mice were used in this study. The Mincle-KO mouse line was obtained from the NIH-sponsored Mutant Mouse Regional Resource Center National System and was back-crossed onto the C57BL/6 background for 10 generations. FcRγ (Fcer1g)-KO mice on a C57BL/6 background (model 583) were obtained from Taconic Biosciences. All animal experimental procedures were approved by the Bioethics Committee of the N. F. Gamaleya Federal Research Center of Epidemiology and Microbiology.

We examined atherosclerotic plaques from patients who underwent carotid endarterectomy surgery. We used formalin-fixed, paraffin-embedded tissues as well as plaques which were freshly harvested from patients and subsequently frozen. Human umbilical cords were freshly harvested postpartum. Informed consent was given by patients as well as by both parents before birth, and the studies were approved by the local ethics committee.

HUVECs were purchased from Cell Applications Inc. For colocalization analysis we used the Coloc2 Plugin in Fiji (37) to calculate the Pearson correlation coefficient after threshold adjustment via the Costes method (38). Isolation of mouse brain endothelial cells was performed using MACS technology (39) or using puromycin selection according to a previously published method (40). Quantification of fluorescent GM3 uptake by endothelial cells was performed using CellProfiler 3.1.8 (41).

Additional information is provided in *SI Appendix*, *Materials and Methods*.

## Supplementary Material

Supplementary File

## Data Availability

All study data are included in the article and/or *SI Appendix*.
