# Supplementary material for: A mechanism of self-lipid endocytosis mediated by the receptor Mincle
Source: Proc Natl Acad Sci U S A. 2022 Jul 22;119(30):e2120489119. doi: 10.1073/pnas.2120489119 (PMC9335232; doi:10.1073/pnas.2120489119)
Supplement: Supplementary File [file pnas.2120489119.sapp.pdf]

Supplementary Information

Materials and methods

Mice

C57BL/6 and C57BL/6-Mincle-KO mice of both sexes were used in this study. The Mincle-KO mouse line was obtained from the National Institutes of Health-sponsored Mutant Mouse Regional Resource Center (MMRRC) National System and was back-crossed onto the C57BL/6 background for 10 generations. FcRγ (Fcer1g)-KO mice on a C57BL/6 background (model 583) were obtained from Taconic Biosciences (Rensselaer, NY). All mice were between 8 wk and 10 wk of age. Mice were fed a completely pelleted laboratory chow and had access to food and water *ad libitum*. All animal experimental procedures were approved by the Bioethics Committee of the N. F. Gamaleya Federal Research Center of Epidemiology and Microbiology.

Histological and immunohistochemical staining of human specimens

We examined 6 atherosclerotic plaques from patients who underwent carotid endarterectomy surgery. We used formalin-fixed, paraffin-embedded tissues that was left from early published study [1], and the protocol was approved by the local ethics committee. All the participants provided written informed consent. Human umbilical cords (n=6) were freshly harvested postpartum and flushed with saline to remove blood from the umbilical vein; a piece of each cord was fixed with 4% (v/v) buffered formalin solution and embedded in paraffin. Informed consent was given by both parents before birth, and the study was approved by the local ethics committee. Paraffin blocks were cut into 4-μm sections. Serial 4-μm sections of formalin-fixed atherosclerotic plaque or umbilical cord tissue were sliced, and then were stained with Caracci's haematoxylin and eosin or were used for immunohistochemical studies. For antigen retrieval, sections were heated in 10 mM sodium citrate buffer (pH 6.0) for 20 min. Next, samples were incubated with blocking buffer (3% (w/v) bovine serum albumin (BSA), 0.1% (w/v) Tween 20 in phosphate-buffered saline (PBS)) for 30 min at 37 °C and stained with primary antibodies overnight at 4 °C. The following primary antibodies were used: anti-Mincle (1:50, sc390806, Santa Cruz Biotechnology) and anti-CD31 (1:50, NB100-2284, Novusbio). After washing, the samples were incubated with AlexaFluor 555- and 594-conjugated secondary antibodies (1:500, A-31570 and A-21207, Thermo Fisher Scientific) for 30 min at 37 °C. Next, cell nuclei were counterstained with DRAQ5 (1:1,000, Thermo Fisher Scientific).

In additional experiments atherosclerotic plaques (n=3) freshly harvested from patients who underwent carotid endarterectomy surgery was frozen, the specimens

were placed in an OCT compound (Tissue-Tek, Elkhart, IN), cut to a 5-µm sections using cryotome (Leica CM 1860 UV, Leica Microsystems), and mounte onto adhesive micro slides (Surgipath X-tra, Leica Microsystems). Then slides were stained with Caracci’s haematoxylin and eosin or were used for immunohistochemical studies. Immunohistochemical staining were performed as described above, only the antigen retrieval operation was omitted. The protocol was approved by the local ethics committee, and all the participants provided written informed consent.

Microscopic images of haematoxylin/eosin stained sections were obtained using a Keyence microscope (BZ-9000). For immunohistochemical studies, samples were imaged using a confocal microscope (TCS SP5 STED, Leica Microsystems, 40× oil immersion objective). Deconvolution was performed in LAS AF software (Leica Microsystems).

HUVEC cultivation

Human umbilical vein endothelial cells (HUVECs) were purchased from Cell Applications Inc., San Diego, CA (cat. number 200p-05n). The cells were resuspended in endothelial basal medium-2 (cc-3156, Lonza) that was supplemented with the Endothelial Cell Growth Medium (EGM)-2 BulletKit (cc-3162, Lonza), and seeded into 6-well plates. Cells were cultured in a humidified atmosphere containing 5% (v/v) CO_2 at 37 °C. The confluent primary monolayers were washed and trypsinized (0.05% (w/v) trypsin + 0.02% (w/v) EDTA, Gibco). Cells were resuspended in complete medium, seeded on 24-well plates (approximately 150,000 cells/mL) with or without coverslips, and cultured for one day. Only the first passage of subcultured cells was used for experiments.

HUVEC immunofluorescence

One coverslip (diameter 12 mm, thickness 0.170 ± 0.005 mm; CG15NH1, Thorlabs) was placed in wells of 24-well culture plates. HUVECs were seeded on the coverslips in endothelial basal medium-2 (Lonza cc-3156) that was supplemented with the Endothelial Cell Growth Medium (EGM)-2 BulletKit (cc-3162; Lonza) and cultured in an atmosphere containing 5% (v/v) CO2 at 37 °C until a monolayer formed. Then, the medium was removed, and after three washes with PBS, cells were fixed with 4% (v/v) buffered formaldehyde at room temperature for 15 min. After three washes with PBS for 5 min each, the fixed cells were permeabilized and blocked with PBS supplemented with 3% (w/v) bovine serum albumin, 0.5% (w/v) Triton X-100 (Amresco) and 0.1% (w/v) Tween 20 (Amresco) for 30 min at 37 °C. Then, the cells were incubated with primary antibodies against Mincle (1:50, sc390806, Santa Cruz Biotechnology), CD31 (1:50, NB100-2284, Novusbio), Clathrin (1:50, 4796, Cell Signaling Technology), Caveolin-1 (1:400, 3267, Cell Signaling Technology), RCAS1 (1:200, 12290, Cell Signaling Technology),

LAMP1 (1:200, 9091, Cell Signaling Technology) in PBS containing 3% bovine serum albumin and 0.1% Tween 20 at 4 °C overnight. After rinsing with PBS three times for 5 min each, cells were incubated with AlexaFluor 555- and 488-conjugated secondary antibodies (1:500, A-31570 and A-21206, Thermo Fisher Scientific) in PBS containing 3% (w/v) bovine serum albumin and 0.1% (w/v) Tween 20 in the dark for 30 min at 37 °C. Finally, after rinsing with PBS three times, nuclei were counterstained with DAPI (Sigma-Aldrich). The coverslips were mounted in Mowiol (containing 6 g of glycerol (Sigma-Aldrich), 2.4 g of Mowiol 4-88 (Sigma-Aldrich), 6 mL of water (obtained from a Milli-Q Advantage A10 system, Millipore), 12 mL of 0.2 M Tris buffer (pH 8.5), and 52.8 mg of 1,4-diazabicyclo[2,2,2]octane (Dabco; Sigma-Aldrich)) and imaged via confocal microscopy (TCS SP5 STED, Leica Microsystems, 40× oil immersion objective; or Nikon N-SIM, Nikon 100×/1.49 NA oil immersion objective). Deconvolution was performed in LAS AF software (Leica Microsystems) for confocal images or NIS Elements software (Nikon) for SIM images.

Western blot analysis

Cells were detached using Trypsin-EDTA, washed twice in ice-cold PBS, centrifuged, and resuspended in ice-cold RIPA buffer (89900, Pierce) containing a complete protease inhibitor (Roche Diagnostics). Approximately 0.5 mL of the buffer was used per 2×10^5 cells. Sample homogenates were prepared using a FastPrep 24 instrument and tubes containing Lysing Matrix A (all from MP Biomedicals). The homogenates were centrifuged at 12,000 × g for 12 min at 4 °C. Next, the extracted proteins and molecular weight markers (ab115832, Abcam) were separated via SDS-PAGE and transferred to nitrocellulose membranes. Samples from HUVECs were reacted with antibodies against Mincle (clone 1H2, D 360-3, MBL International, 1:1,000 or clone E5, sc390807, Santa Cruz Biotechnology, 1:200). Samples from mouse brain endothelial cells were reacted with antibodies against Mincle (clone B7, sc390806, Santa Cruz Biotechnology, 1:200). Detection was performed using an HRP-conjugated goat anti-rat IgG antibody (1:10,000, NA935V, GE Healthcare) or a goat anti-mouse IgG antibody (1:1,000, HAF007, R&D Systems), and bands were visualized using an Optiblot ECL Max Detect Kit (ab133408, Abcam) according to the provided protocol.

Flow cytometry analysis of human and mouse endothelial cells

Flow cytometry analysis was performed using a MACSQuant Analyzer 10 flow cytometer equipped with three laser excitation sources (405 nm, 488 nm, and 635 nm), and the data were evaluated using MACSQuantify V2.11.1817.19623 software (all from Miltenyi Biotec, Germany). Staining was performed with the following fluorochrome-conjugated antibodies: anti-mouse CD31-PE (1:50, 130-111-354),

anti-mouse CD31-APC (1:50, 130-111-355), anti-mouse CD45-APC-Vio 770 (1:50, 130-110-662), anti-human CD31-PE (1:50, 130-110-669), anti-human CD45-APC-Vio 770 (1:50, 130-110-635, all from Miltenyi Biotec), and anti-human/mouse Mincle-AlexaFluor 546 (1:50, sc-390806 AF546, Santa Cruz Biotechnology). Cells were labelled with antibodies for 10 min and washed with PBS containing 1% (v/v) foetal bovine serum. Then, 7-AAD dye (Miltenyi Biotec) was added to the suspension of live cells just before flow cytometric analysis.
For intracellular staining, live cells were labelled with anti-mouse CD31-APC (1:50, 130-111-355, Miltenyi Biotec) for 10 min, washed with PBS containing 1% (v/v) foetal bovine serum and permeabilized with Cytofix/Cytoperm solution (BD Biosciences) for 20 min according to the manufacturer's instructions. Intracellular labelling was performed with the mouse anti-Mincle (Clec4e)-AlexaFluor 546 (sc-390806 AF546, Santa Cruz Biotechnology) antibody for 20 min, followed by two washes with Perm-Wash buffer and flow cytometry analysis.

Data were generated by flow cytometry analysis of more than 20,000 events. The percentages of each positive population and mean fluorescence intensity (MFI) values were determined using quadrant statistics. Gating strategies are depicted in Fig. S10 (HUVECs) and Fig. S11 (mouse brain endothelial cells).

Super-resolution microscopy (SIM)

Samples were imaged using the Nikon N-SIM microscope (Nikon, Japan; 100×/1.49 NA oil immersion objective, 488 nm and 561 nm diode laser excitation). Image stacks (z-steps of 0.12 μm) were acquired with an iXon 897 EMCCD camera (Andor Technology, UK) with an effective pixel size of 60 nm. Exposure conditions were adjusted to obtain a typical yield of approximately 5,000 max counts (16-bit raw image) while minimizing bleaching. Image acquisition, SIM image reconstruction data alignment and deconvolution using the standard Richardson-Lucy algorithm were performed in NIS Elements (Nikon).

Measurement of the diameter of intracellular Mincle-containing bodies

HUVECs stained for Mincle were used in this experiment. Samples were imaged using the Nikon N-SIM microscope (Nikon, Japan; 100×/1.49 NA oil immersion objective, 488 nm and 561 nm diode laser excitation), SIM image reconstruction and data alignment were performed, and the images were then deconvoluted using the standard Richardson-Lucy algorithm. Then, the data were imported into Imaris 7.2 software (Bitplane, Inc.). The diameters of Mincle-containing bodies were measured using the Imaris Spot tool. In total, five cells were analysed (14,768 measurements). The data were imported into Microsoft Excel to construct a histogram.

Identification of Mincle in HUVEC cell lysates by mass spectrometry

HUVECs (10^6) were detached using Trypsin-EDTA, washed twice in ice-cold PBS containing a complete protease inhibitor (Roche Diagnostics), centrifuged, and dried in a vacuum concentrator (Eppendorf, Germany). Then, 30 µL of lysis buffer (4% (w/v) SDS, 100 mM β-mercaptoethanol) was added to the dried cells following incubation at 4 °C for 24 hours, followed by boiling for 5 min before separation. Proteins were separated in a Mini PROTEAN Tetra gel electrophoresis system (BioRad) on a 15% (w/v) polyacrylamide gel (via PAGE). Excised gel bands corresponding to proteins with masses of 25-30 kDa were digested with trypsin (Promega). After lyophilization, tryptic peptides were analysed by LC-MS. The LC-MS apparatus consisted of an Easy-nLC 1000 (Thermo Scientific) nano-LC system and an Orbitrap Elite ETD mass spectrometer (Thermo Scientific). Peptides were separated on a custom-made column (75 µm I.D., 150 mm length) packed with 1.7 µm Aeris PEPTIDE XB-C18 beads (Phenomenex). The column effluent was sprayed directly into the interface of the mass spectrometer. The linear gradient used for separation went from 100% (by volume) buffer A (3% (v/v) acetonitrile, 0.05% (v/v) formic acid, 0.05% (v/v) trifluoroacetic acid and 96.9% (v/v) water) to 60% buffer B (80% (v/v) acetonitrile, 0.05% (v/v) formic acid, 0.05% (v/v) trifluoroacetic acid and 19.9% (v/v) water) over 180 min. The capillary entrance of the mass spectrometer was maintained at a temperature of 200 °C. MS spectra were recorded under a resolving power of 60,000, while MS/MS spectra were obtained with a higher-energy collisional dissociation (HCD) cell and registered under a resolving power of 15,000. Raw mass spectrometric data were screened against a custom human protein database derived from the UniProt database using the PEAKS Studio 7.5 search engine (Bioinformatics Solutions). The following search settings were used: parent mass error tolerance, 10.0 ppm; fragment mass error tolerance, 0.1 Da; precursor mass search type, monoisotopic; enzyme, trypsin; max missed cleavages, 3; non-specific cleavage, both; fixed modifications, carbamidomethylation—57.02; variable modifications, oxidation (M)—15.99, formylation—27.99, formylation (protein N-term)—27.99; max number of modifications per peptide, 3.

SPR binding analysis

The SPR experiments were performed using a BIACORE 3000 (GE Healthcare) equipped with a research-grade CM5 sensor chip (BR100012, GE Healthcare) at a temperature of 25 °C. Histidine-tagged recombinant human Mincle (Clec4e) in 10 mM acetate buffer (pH 4.5), derived from human cells (C588, Novoprotein), was covalently immobilized onto the sensor chip surface at a level of ~10,000 response units (RU) using an Amine Coupling Kit (BR-1000-50, GE Healthcare). The

following glycosphingolipids were used in these experiments: C18 lactosyl(ß) ceramide (d18:1/18:0) (860598), ganglioside GM3 (860058), C18 glucosyl(ß) ceramide (d18:1/18:0) (860547), ganglioside GM1 (860065), ganglioside GD3 (860060), lysoglucosyl(ß) ceramide (d18:1) (860535; all from Avanti Polar Lipids), and N-glycolyl-Ganglioside GM3 (Neu5Gc GM3, 33263, Cayman Chemical). The analytes were dissolved in DMSO with heating (60 °C for 5 min). Then, the stock solution was diluted in buffer containing 20 mM HEPES and 150 mM NaCl (pH 7.2), to obtain a 5% (v/v) DMSO concentration in the final solution, and the solution was then vortexed for 1 min. Finally, two rounds of ultrasonication (30 sec each) were conducted using a Branson S-450D instrument to obtain homogenous stock solutions. Serial dilutions were then performed using running buffer (20 mM HEPES, 150 mM NaCl, 5% (v/v) DMSO (pH 7.2)). Analyses were performed at 25 °C in running buffer. The glycoshingolipids were injected at different indicated concentrations and passed over adjacent target and control flow cells at a flow rate of 30 μL/min for 4 min. After dissociation phase, the bound analytes were removed by regeneration buffer (20 mM Tris·HCl, 5% DMSO (v/v) and 0.05% (w/v) Tween 20 (pH 7.2)) which was injected two times (2 min per injection) for chip regeneration. Calculations were performed with double referencing (blank surface and blank buffer referencing) using BIAevaluation software (GE Healthcare, Sweden).

Super-resolution microscopy studies of fluorescently labelled lipid uptake by human endothelial cells

Glucosylceramide (810223P, Avanti Polar Lipids) and lactosylceramide (810227P, Avanti Polar Lipids), both labelled with the NBD fluorescent label , and ganglioside GM3 labelled with TopFluor fluorescent label (810258, Avanti Polar Lipids) were used in this study. As TopFluor-labelled ganglioside GM3 was supplied as an ethanol solution, it was first dried under vacuum. Then, a 0.2% (w/v) aqueous solution of carboxymethylcellulose sodium salt (C9481, Sigma-Aldrich) was added to each of the fluorescent glycosphingolipids, and five rounds of ultrasonication (30 sec each) were conducted using a Branson S-450D instrument to obtain homogenous stock solutions (25 μg/mL). The samples were cooled for three minutes between the rounds of ultrasonication. Working solutions of the fluorescent glycosphingolipids were prepared by diluting the stock solutions to a concentration of 2.5 μg/mL in a complete cell culture medium.

The working solutions of the fluorescent glycosphingolipids were added to HUVECs at a volume of 1 mL. Cells were incubated with glycosphingolipids for 1 hour. Then, the medium was removed, and after three washes with PBS, the cells were fixed with 4% (v/v) buffered formaldehyde at room temperature for 15 min.

After washing three times with PBS for 5 min, the fixed cells were permeabilized and blocked with PBS supplemented with 3% (w/v) bovine serum albumin, 0.5% (w/v) Triton X-100 and 0.1% (w/v) Tween 20 for 30 min at 37 °C. Then, the cells were incubated with AlexaFluor 546-conjugated anti-Mincle primary antibodies (1:10, sc390806, Santa Cruz Biotechnology) in PBS containing 3% (w/v) bovine serum albumin and 0.1% (w/v) Tween 20 at 4 °C overnight. Coverslips were mounted in Mowiol (containing 6 g of glycerol (Sigma-Aldrich), 2.4 g of Mowiol 4-88 (Sigma-Aldrich), 6 mL of water (obtained from a Milli-Q Advantage A10 system, Millipore), 12 mL of 0.2 M Tris buffer (pH 8.5), and 52.8 mg of Dabco (Sigma-Aldrich) and were imaged via super-resolution microscopy (Nikon N-SIM microscope, Nikon 100×/1.49 NA oil immersion objective).

Colocalization analysis

Samples were imaged using the Nikon N-SIM microscope (Nikon, Japan; 100×/1.49 NA oil immersion objective, 488 nm and 561 nm diode laser excitation), SIM image reconstruction and data alignment were performed, and the images were then deconvoluted using the standard Richardson-Lucy algorithm. We used the Coloc2 Plugin in Fiji [2] to calculate the Pearson correlation coefficient after threshold adjustment via the Costes method [3].

Preparation of a single-cell suspension from adult mouse brain tissue

Intact C57BL/6 wild type, C57BL/6-Mincle-KO mice, and FcRγ-KO mice were used. Brains of eight mice per genotype were used for each isolation with MACS, and four or five brains were used for each experiment using puromycin selection. All mice were between 9 and 10 wk old. Mice were euthanized by carbon dioxide inhalation, and the brains were harvested. Brain tissue was dissociated into single-cell suspensions using an Adult Brain Dissociation Kit in gentleMACS C-tubes on a gentleMACS Octo Dissociator with Heaters (all from Miltenyi Biotec) according to the manufacturer's recommended protocol. In brief, 500 mg of brain tissue was transferred into one C-tube containing 1,950 µL of enzyme mix 1. Then, 30 µL of enzyme mix 2 was added to the C-tube, and dissociation was started. The extracellular matrix was enzymatically digested using the kit components, and the samples were passed through a 100 µm cell strainer. Then, the cell strainer was washed with 10 mL of cold PBS containing calcium and magnesium and supplemented with glucose and sodium pyruvate (Capricorn Scientific), and the resulting single-cell suspension was centrifuged at 300 × g for 10 min. Myelin and cell debris were removed using Debris Removal Solution (Miltenyi Biotec), and erythrocytes were removed using Red Blood Cell Removal Solution (Miltenyi Biotec). The quantity of endothelial cells in the single-cell suspension from the

brains from Mincle-KO mice and wild-type mice was assessed by flow cytometry using anti-CD31-PE (130-111-354) and anti-CD45-APC-Vio 770 (130-110-662, all from Miltenyi Biotec) antibodies. The gating strategy is depicted in Fig. S11.

MACS isolation and subsequent culture of mouse brain endothelial cells

Isolation of endothelial cells was performed using MACS technology [4]. Endothelial cells obtained from single-cell suspensions from the brains of Mincle-KO mice and wild-type mice were enriched by depletion of CD45+ cells with CD45 MicroBeads followed by a positive selection step using CD31 MicroBeads (all from Miltenyi Biotec). Approximately 2×10^7 cells were resuspended in 180 µL of cold separation buffer (PBS containing 0.5% (w/v) bovine serum albumin, PBS/BSA), incubated for 15 min at 4 °C with 20 µL of mouse CD45 MicroBeads (130-052-301, Miltenyi Biotec), washed by adding 2 mL of PBS/BSA separation buffer and centrifuged at 300 × g for 5 min. Cells were resuspended in 1 mL of PBS/BSA separation buffer and applied to prepared MACS columns (LD type; Miltenyi Biotec) placed in the magnetic field of a MidiMACS Separator (Miltenyi Biotec) according to the manufacturer's recommended protocol. Unlabelled cells that passed through the column were collected and used as CD45- cells in the second stage of positive selection. The CD45- fraction was centrifuged at 300 × g for 5 min, resuspended in 180 µL of cold PBS/BSA separation buffer, incubated with 20 µL of mouse CD31 MicroBeads (130-097-418, Miltenyi Biotec), washed by adding 2 mL of PBS/BSA separation buffer and centrifuged at 300 × g for 5 min. Cells were resuspended in 500 µL of separation buffer and applied to a MACS column (MS type; Miltenyi Biotec) placed in the magnetic field of a MiniMACS Separator (Miltenyi Biotec). Then, the column was washed three times with 500 µL of separation buffer, and the retained magnetically labelled cells were eluted with 1 mL of separation buffer as the positive fraction. Aliquots of the positive fraction were used for western blotting and flow cytometry. The quality of endothelial cells after separation was assessed by flow cytometry using anti-CD31 (1:50, 130-111-354, CD31-PE, Miltenyi Biotec) and anti-CD45 (1:50, 130-110-662 CD45-APC-Vio 770, Miltenyi Biotec) antibodies. The gating strategy is depicted in Fig. S11.

To culture the isolated endothelial cells, coverslips (diameter 12 mm, thickness 0.170 ± 0.005 mm; CG15NH1, Thorlabs) were placed in wells of 24-well culture plates and pre-coated with collagen (C8919, Sigma-Aldrich) and fibronectin (F1141, Sigma-Aldrich) at a ratio of 3 µg each protein per cm^2, incubated overnight at 37 °C and washed three times with ddH_2O. After magnetic separation, endothelial cells were resuspended in culture medium (EndoPrime Complete Medium, Capricorn Scientific) and plated on coverslips (10^5 cells per coverslip). After 24 hours of culture in 5% (v/v) CO_2, non-adherent cells were removed from the coverslips by

replacement of the culture medium. Endothelial cells were cultured for 2 days and were then used for confocal microscopy and super-resolution microscopy (SIM) experiments.

Puromycin selection isolation and subsequent culture of mouse brain endothelial cells

The single-cell suspensions obtained from the brains of Mincle-KO mice, FcRγ-KO mice and wild-type mice were cultured in the presence of puromycin according to a previously published method [5]. Cells were resuspended in EndoPrime complete medium (Capricorn Scientific) containing puromycin (InvivoGen) at a concentration of 8 μg/mL and seeded in wells of 24-well plates pre-coated with collagen (C8919, Sigma) and fibronectin (F1141, Sigma-Aldrich). For other experiments, cells were seeded on coverslips (diameter 12 mm, thickness 0.170 ± 0.005 mm; CG15NH1, Thorlabs), which were placed in wells of 24-well culture plates and pre-coated with collagen (C8919, Sigma-Aldrich) and fibronectin (F1141, Sigma-Aldrich). On the second day of culture, cells were washed twice with calcium- and magnesium-free Dulbecco's PBS (Capricorn Scientific), and fresh EndoPrime complete medium containing puromycin. On the third day, the medium was replaced with fresh EndoPrime complete medium without added puromycin. Cells were cultured further until a monolayer formed (to confluence), with replacement of the medium every 2 days. Monolayer cell cultures were used for confocal microscopy and flow cytometry experiments. For flow cytometric analysis, cells from Mincle-KO mice and wild-type mice were used. Cells were detached from the wells of the plates or from the coverslips with 0.05% (w/v) trypsin-EDTA solution. The quality of endothelial cells after separation was assessed by flow cytometry using anti-CD31 (1:50, 130-111-354, CD31-PE, Miltenyi Biotec) and anti-CD45 (1:50, 130-110-662 CD45-APC-Vio 770, Miltenyi Biotec) antibodies. The gating strategy is depicted in Fig. S11. As EndoPrime complete medium (Capricorn Scientific) now is discontinued, in some experiments we used Complete Mouse Endothelial Cell Medium (M1168, Cell Biologics, IL) instead EndoPrime medium.

Immunofluorescence analysis of mouse brain endothelial cells

Mouse brain endothelial cells were cultured on coverslips (diameter 12 mm, thickness 0.170 ± 0.005 mm; CG15NH1, Thorlabs), which were placed in wells of 24-well culture plates and pre-coated with collagen (C8919, Sigma) and fibronectin (F1141, Sigma-Aldrich). After cultivation, the medium was removed, and after three washes with PBS, cells were fixed with 4% (v/v) buffered formaldehyde at room temperature for 15 min. After three washes with PBS for 5 min each, the fixed cells

were permeabilized and blocked with PBS supplemented with 3% (w/v) bovine serum albumin, 0.5% (w/v) Triton X-100 (Amresco) and 0.1% (w/v) Tween 20 (Amresco) for 30 min at 37 °C. Then, the cells were incubated with primary antibodies against Mincle (1:50, sc390806, Santa Cruz Biotechnology) or with primary antibodies against Mincle (1:50, sc390807, Santa Cruz Biotechnology) and CD31 (1:50, NB100-2284, Novusbio) in PBS containing 3% (w/v) bovine serum albumin and 0.1% (w/v) Tween 20 (Amresco) at 4 °C overnight. After rinsing with PBS three times for 5 min, the cells were incubated with AlexaFluor 555- and 488-conjugated secondary antibodies (1:500, A-31570, A-21206, Thermo Fisher Scientific) in PBS containing 3% (w/v) bovine serum albumin and 0.1% (w/v) Tween 20 in the dark for 30 min at 37 °C. Finally, after rinsing with PBS three times, nuclei were counterstained with DAPI (Sigma-Aldrich). The coverslips were mounted in Mowiol (containing 6 g of glycerol (Sigma-Aldrich), 2.4 g of Mowiol 4-88 (Sigma-Aldrich), 6 mL of water (obtained from a Milli-Q Advantage A10 system, Millipore), 12 mL of 0.2 M Tris buffer (pH 8.5), and 52.8 mg Dabco (Sigma-Aldrich)) and imaged via confocal microscopy (TCS SP5 STED, Leica Microsystems, 40× oil immersion objective; or Nikon N-SIM microscope, Nikon 100×/1.49 NA oil immersion objective). Deconvolution was performed in LAS AF software (Leica Microsystems) for confocal images or NIS Elements software (Nikon) for SIM images.

Confocal microscopy studies of fluorescently labelled GM3 ganglioside uptake by mouse brain endothelial cells

Fluorescent TopFluor-labelled ganglioside GM3 (810258, Avanti Polar Lipids) was used in this study. As the reagent was supplied as an ethanol solution, it was dried under vacuum, dissolved in PBS, and subjected to ultrasonication for 30 sec using the Branson S-450D instrument to obtain a 40 µg/mL stock solution. The GM3 ganglioside working solution was prepared by diluting the stock solution in EndoPrime complete medium (Capricorn Scientific) to a concentration of 2 µg/mL. A 300 µL volume of GM3 ganglioside working solution was added to cultured endothelial cells isolated from the brains of Mincle-KO, FcRγ-KO mice or wild-type mice using puromycin selection or MACS (the cells were seeded on coverslips (CG15NH1, Thorlabs)).Cells were incubated with the ganglioside solution for 2 hours. Then, the medium was removed, and after three washes with PBS buffer (prepared from a tablet; P4417, Sigma-Aldrich), cells were fixed with 4% (v/v) buffered formaldehyde at room temperature for 15 min. After washing three times with PBS buffer for 5 min each, the fixed cells were blocked with PBS supplemented with 3% (w/v) bovine serum albumin and 0.1% (w/v) Tween 20 for 30 min. To stain cell boundaries, monolayer cultures of cells isolated using puromycin selection were

incubated with primary antibodies against CD31 (1:50, NB100-2284, Novusbio) in PBS containing 3% (w/v) bovine serum albumin and 0.1% (w/v) Tween 20 at 4 °C overnight. After rinsing with PBS three times for 5 min, cells were incubated with AlexaFluor 594-conjugated secondary antibodies (1:500, A-21207, Thermo Fisher Scientific) in PBS containing 3% (w/v) bovine serum albumin and 0.1% (w/v) Tween 20 in the dark for 30 min at 37 °C. Brain endothelial cells isolated using MACS were stained with AlexaFluor 647-conjugated phalloidin (1:500, Thermo Fisher Scientific) for 1 hour to delineate their boundaries.

Coverslips were mounted in Mowiol (containing 6 g of glycerol (Sigma-Aldrich), 2.4 g of Mowiol 4-88 (Sigma-Aldrich), 6 mL of water (obtained from a Milli-Q Advantage A10 system, Millipore), 12 mL of 0.2 M Tris buffer (pH 8.5), and 52.8 mg of Dabco (Sigma-Aldrich)) and imaged via confocal microscopy (TCS SP5 STED, Leica Microsystems, 40× or 100× oil immersion objective) to quantify the uptake of fluorescent GM3.

Quantification of fluorescent GM3 uptake by endothelial cells isolated from Mincle-deficient mice and wild-type mice using MACS by confocal microscopy

Deconvolution of acquired confocal images was performed in LAS AF software (Leica Microsystems). Image z-stacks were imported into CellProfiler 3.1.8 [6], where cell boundaries were revealed by staining with AlexaFluor 647-conjugated phalloidin (1:500, Thermo Fisher Scientific), and the number of green pixels (lipid signal) inside the cell boundary was calculated in every optical section. The data are presented as the number of green pixels per cubic micrometre.

Statistical analysis

All experiments were repeated at least twice. Unless stated otherwise, the statistical significance of the differences among the means was determined via an unpaired two-tailed Student's t-test in GraphPad Prism software (GraphPad Software Inc.). Differences were considered significant if P was less than 0.05.

References

1. Lebedeva, A., et al. Ex vivo culture of human atherosclerotic plaques: A model to study immune cells in atherogenesis. *Atherosclerosis*, *267*, 90-98 (2017).
2. Schindelin, J., et al. Fiji: an open-source platform for biological-image analysis. Nature methods. 9, 676-682 (2012).
3. Costes, S., et al. Automatic and quantitative measurement of protein-protein colocalization in live cells. Biophysical Journal. 86, 3993-4003 (2004).

4. Dudek, K. A., et al. Molecular adaptations of the blood–brain barrier promote stress resilience vs. depression. Proceedings of the National Academy of Sciences. 117, 3326-3336 (2020).
5. Assmann, J. C., et al. Isolation and cultivation of primary brain endothelial cells from adult mice. Bio-protocol. 7, e2294 (2017).
6. Carpenter, A., et al. Cell Profiler: image analysis software for identifying and quantifying cell phenotypes. Genome Biol. 7, R100 (2006).

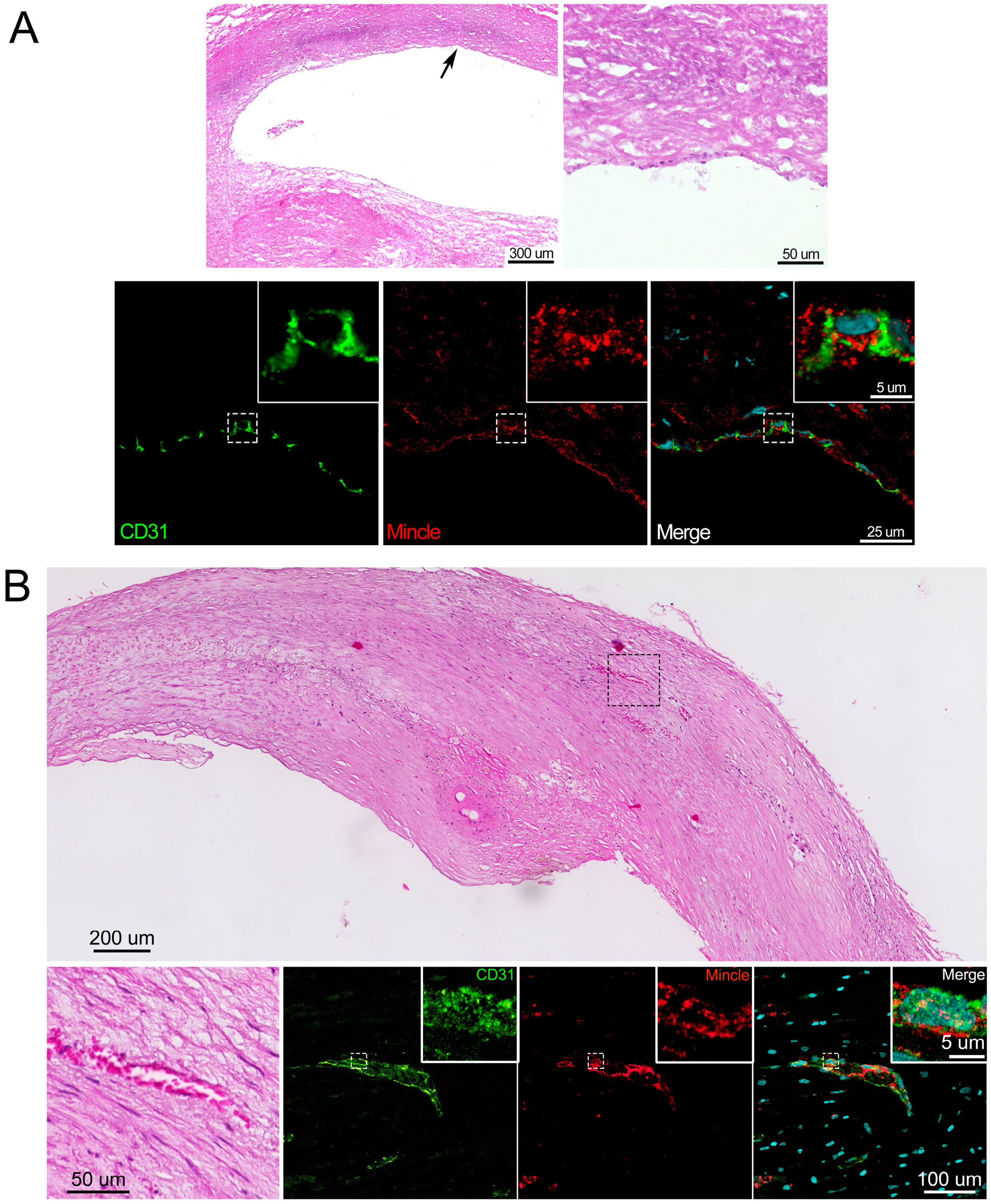

Supplementary Fig. 1. Mincle staining in human carotid plaque specimens collected immediately after endarterectomy.

A. Mincle staining in the endothelium of frozen carotid plaque specimens.

B. Mincle staining in the endothelium of the *vasa vasorum* (formalin-fixed, paraffin-embedded tissues).

Haematoxylin/eosin and immunohistochemical staining using antibodies against Mincle (red) and CD31 (green). Nuclei were costained with DAPI (cyan).

A

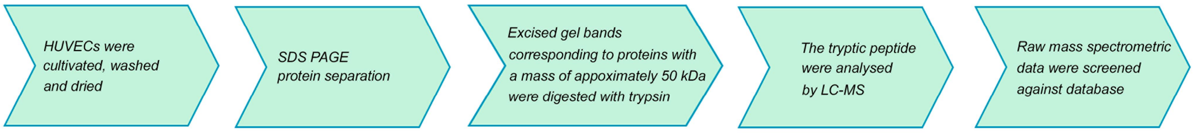

B

Protein Group	Protein ID	Accession	Score (%)	-10lgP	Coverage (%)	#Peptides	#Unique	PTM	Avg. Mass	Description
**2**	1	#DECOY#XP_011518917.1	98.4	175.96	11	182	181	Y	1725124	C-type lectin domain family 4 member E isoform X2 [Homo sapiens]
**1**	3	#DECOY#NP_055173.1	98.1	170.15	9	185	185	Y	2164814	C-type lectin domain family 4 member E [Homo sapiens]
**3**	2	#DECOY#XP_011518916.1	98.7	157.92	8	153	152	Y	1865611	C-type lectin domain family 4 member E isoform X1 [Homo sapiens]
**4**	4	XP_011518916.1	87.1	41.74	20	4	4	Y	21593	C-type lectin domain family 4 member E isoform X1 [Homo sapiens]
**total 4 proteins**										

Supplementary Fig. 2. Mass spectrometry identification of Mincle in HUVEC cell lysates.

A. Schematic representation of the experimental procedure. Proteins in HUVEC lysates were separated on polyacrylamide gels under reducing conditions, excised gel bands corresponding to proteins with a mass of approximately 50 kDa were digested with trypsin, and the tryptic peptides were then analysed by LC-MS. B. Raw mass spectrometric data were screened against a custom human protein database derived from the UniProt database using the PEAKS Studio 7.5 search engine. Mincle was identified by the presence of 4 peptides.

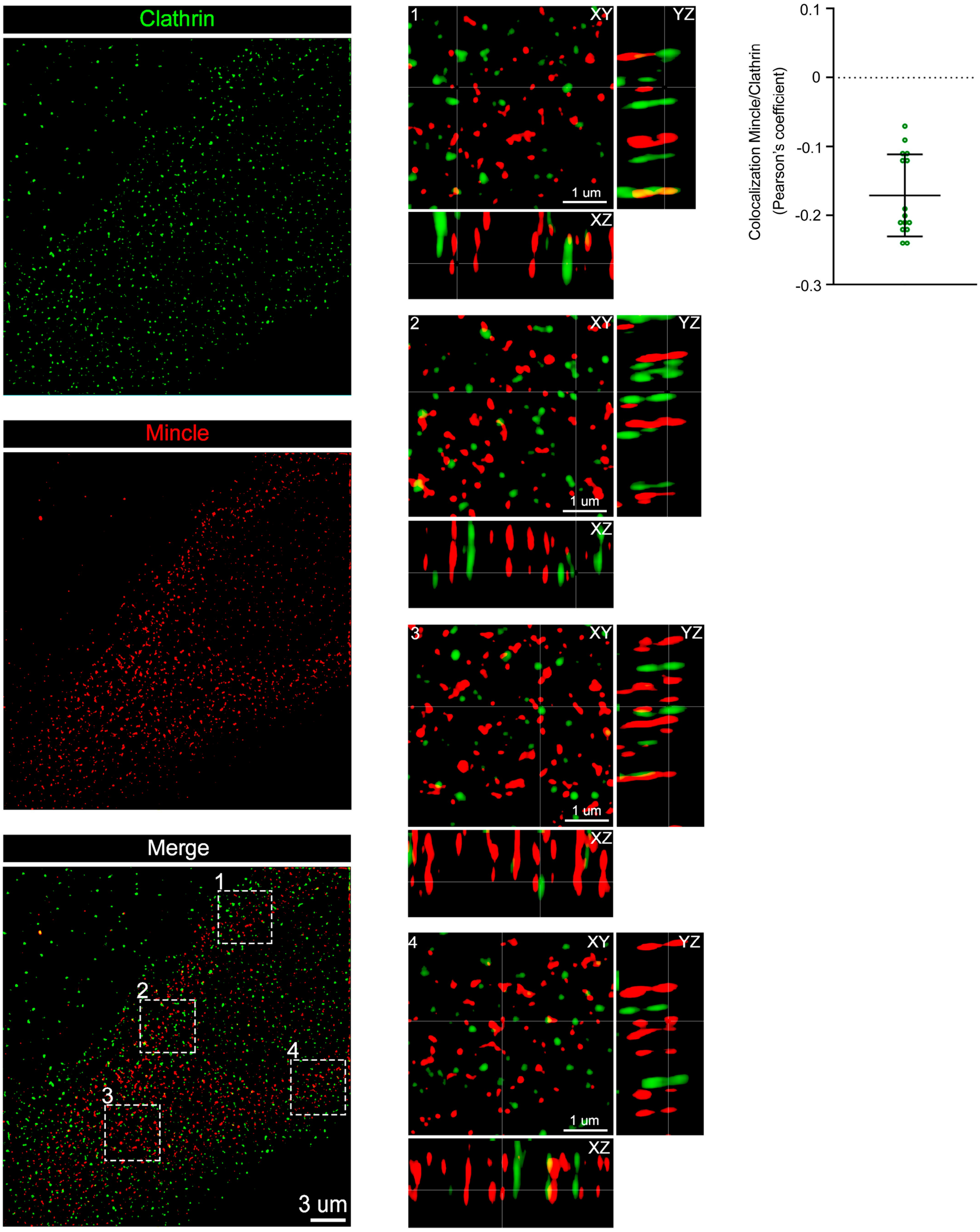

Supplementary Fig. 3. Mincle does not colocalize with clathrin in intact HUVECs.

Left, Intact HUVECs were washed, fixed, stained using antibodies to Mincle (red) and clathrin (green) and analysed by SIM. Representative cells are shown. Two independent experiments were performed.

Right, Statistical analyses of clathrin and Mincle colocalization using the Pearson correlation coefficient; n = 15 independent fields. The results are presented as the mean ± s.d. values.

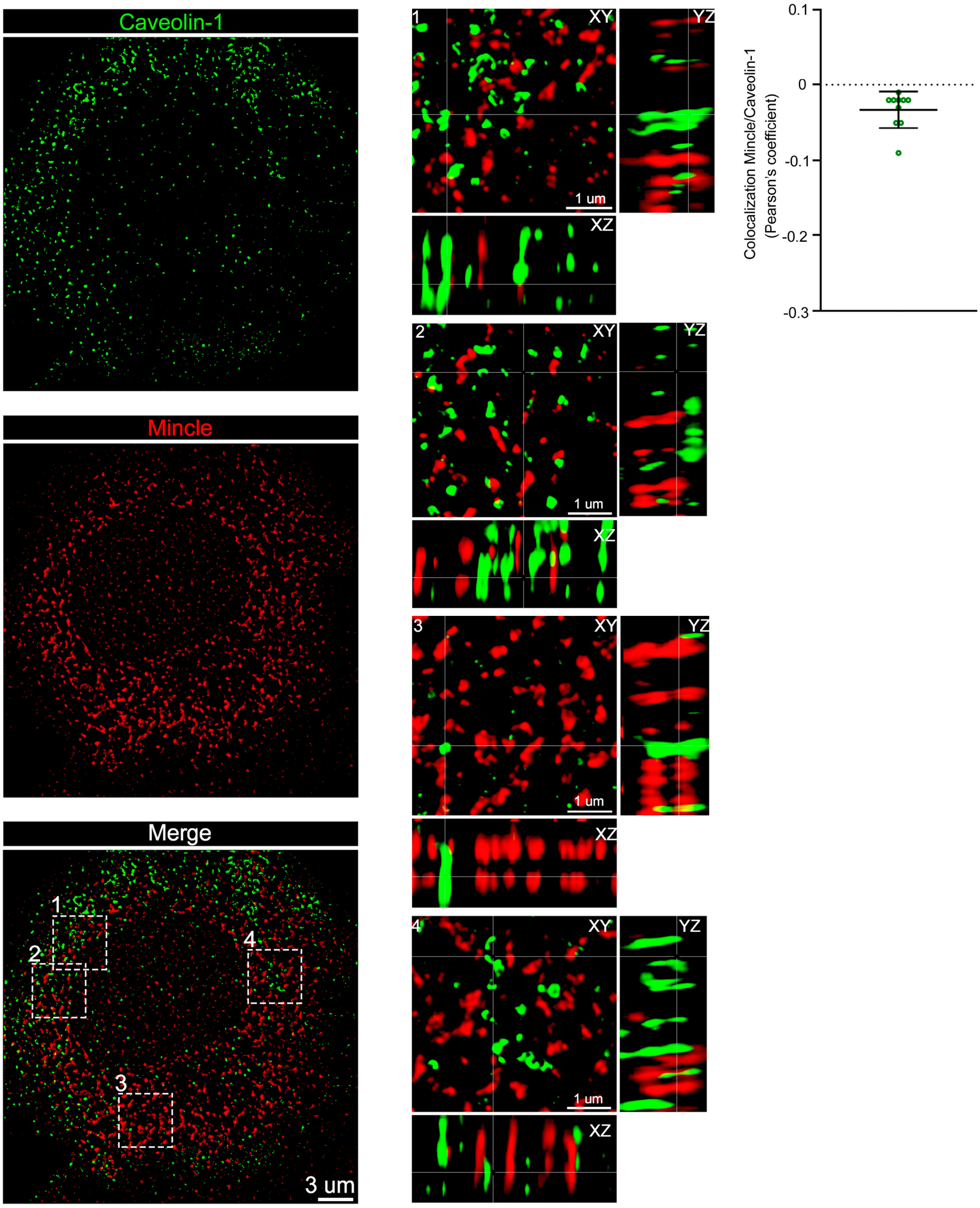

Supplementary Fig. 4. Mincle does not colocalize with caveolin-1 in intact HUVECs.

Left, Intact HUVECs were washed, fixed, labelled with antibodies against Mincle (red) and caveolin-1 (green) and analysed by SIM. Representative cells are shown. Two independent experiments were performed.

Right, Statistical analyses of caveolin-1 and Mincle colocalization using the Pearson correlation; n = 15 independent fields. The results are presented as the mean ± s.d. values.

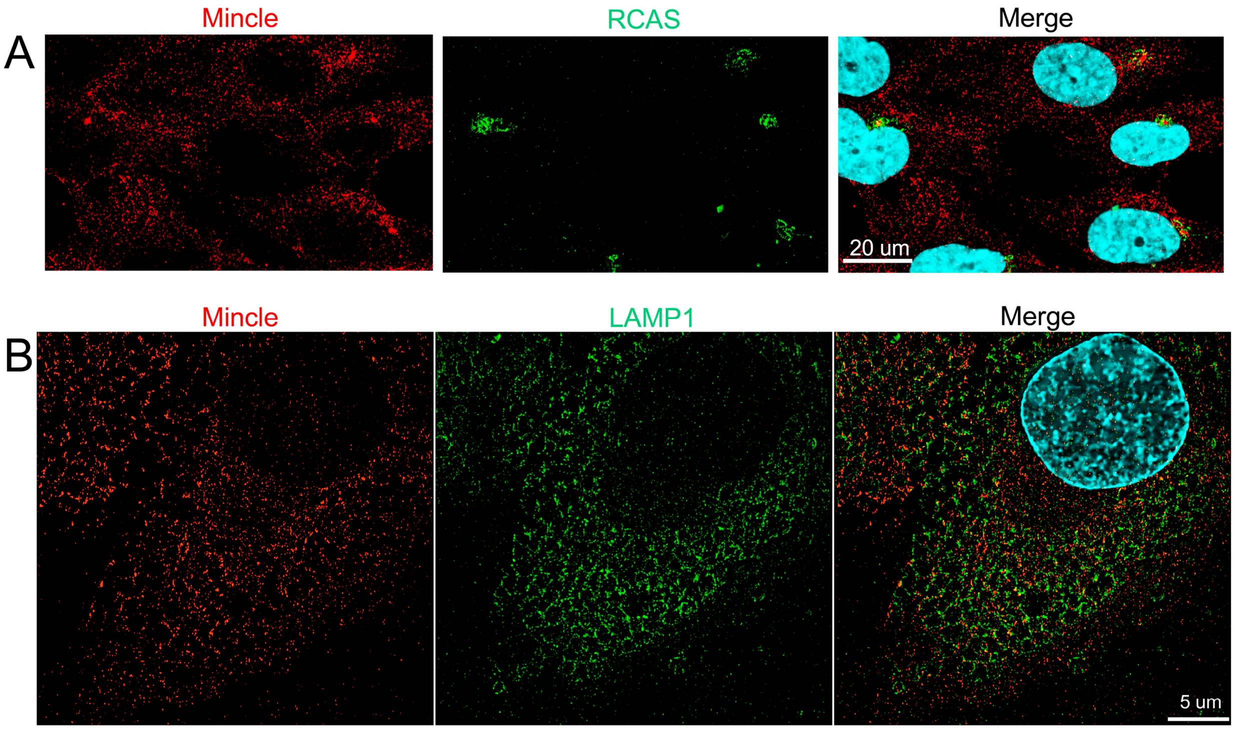

Supplementary Fig. 5. Evaluation of subcellular Mincle localization.

A.HUVECs were costained for Mincle (red) and RCAS1 (green) and imaged by confocal microscopy. Nuclei were costained with DAPI. Representative images are shown. RCAS1 is a Golgi protein with the ability to regulate vesicle formation and secretion.

B.HUVECs were costained for Mincle (red) and LAMP1 (green) and imaged by super-resolution microscopy (SIM). Nuclei were costained with DAPI. Representative images are shown. LAMP1 resides primarily across lysosomal membranes.

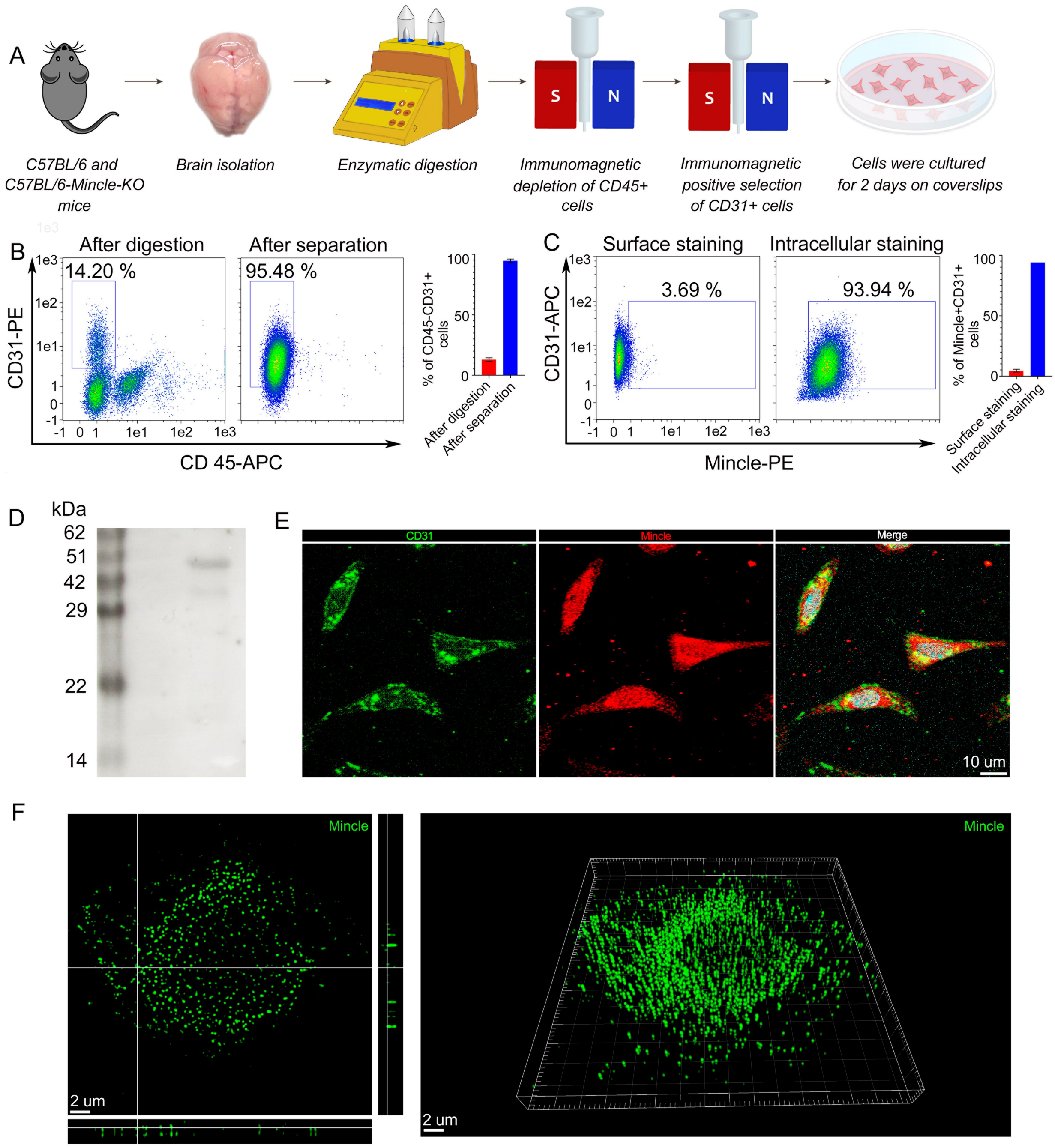

Supplementary Fig. 6. Isolation of endothelial cells from mouse brains by MACS. A. Schematic representation of the brain endothelial cell isolation process. After brain harvesting, enzymatic digestion were performed to obtain a single-cell suspension. MACS was then performed with initial immunomagnetic depletion of CD45+ cells followed by immunomagnetic positive selection of CD31+ cells. B. Flow cytometry was performed on cells stained for CD45 and CD31 after mechanical/enzymatic digestion and after immunomagnetic separation. Flow cytometry plots and quantification of CD45-CD31+ cell percentages are depicted. C. Flow cytometry was performed on cells stained for surface and intracellular Mincle and CD31. Flow cytometry plots and quantification of Mincle+CD31+ cell percentages are depicted.

D. Cell lysates were evaluated for Mincle expression by western blotting. E. Mouse endothelial cells immunomagnetically sorted after 2 days of culture were costained for Mincle (red) and CD31 (green) and imaged by confocal microscopy; nuclei were stained with DAPI (cyan). F. Super-resolution microscopy (SIM) of mouse brain endothelial cells stained for Mincle (green) revealed the presence of this receptor in the cytoplasm in small, uniformly sized bodies, similar to its localization in human endothelial cells (see Fig. 1G).

Representative results from at least two independent experiments are shown in the figure. The results are presented as the mean ± s.d. values.

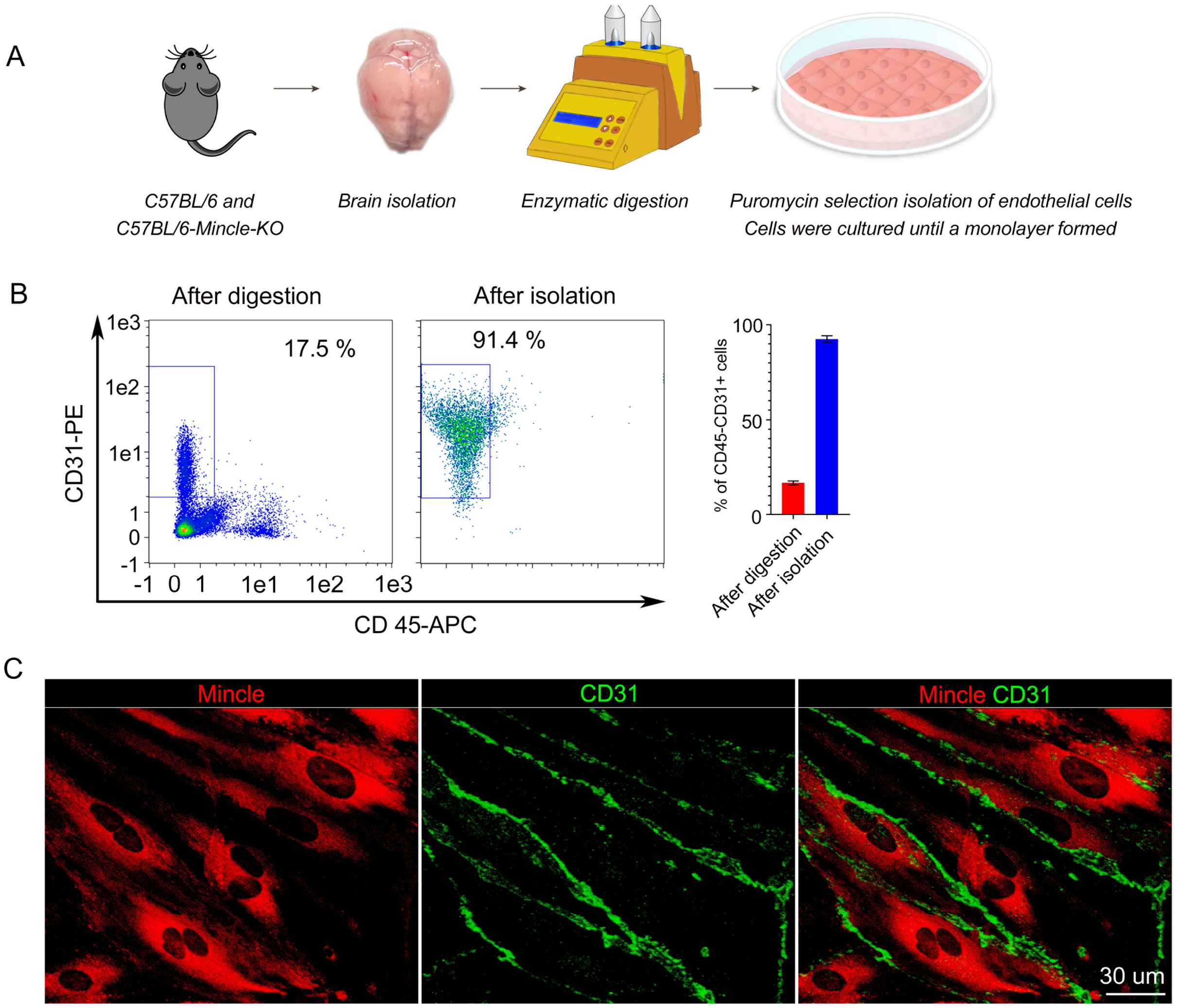

Supplementary Fig. 7. Isolation of endothelial cells from mouse brains using puromycin selection.

A. Schematic representation of the process for brain endothelial cell isolation and selection. After brain isolation, enzymatic digestion were performed to obtain a single-cell suspension, and cells were then cultured in the presence of puromycin until a monolayer formed. B. Flow cytometry was performed on cells stained for CD45 and CD31 after mechanical/enzymatic digestion and after puromycin selection. Flow cytometry plots and quantification of CD45-CD31+ cell percentages are depicted

C. Monolayers of brain endothelial cells were costained for Mincle (red) and CD31 (green) and imaged by confocal microscopy.

Representative results from at least two independent experiments are shown in the figure. The results are presented as the mean ± s.d. values.

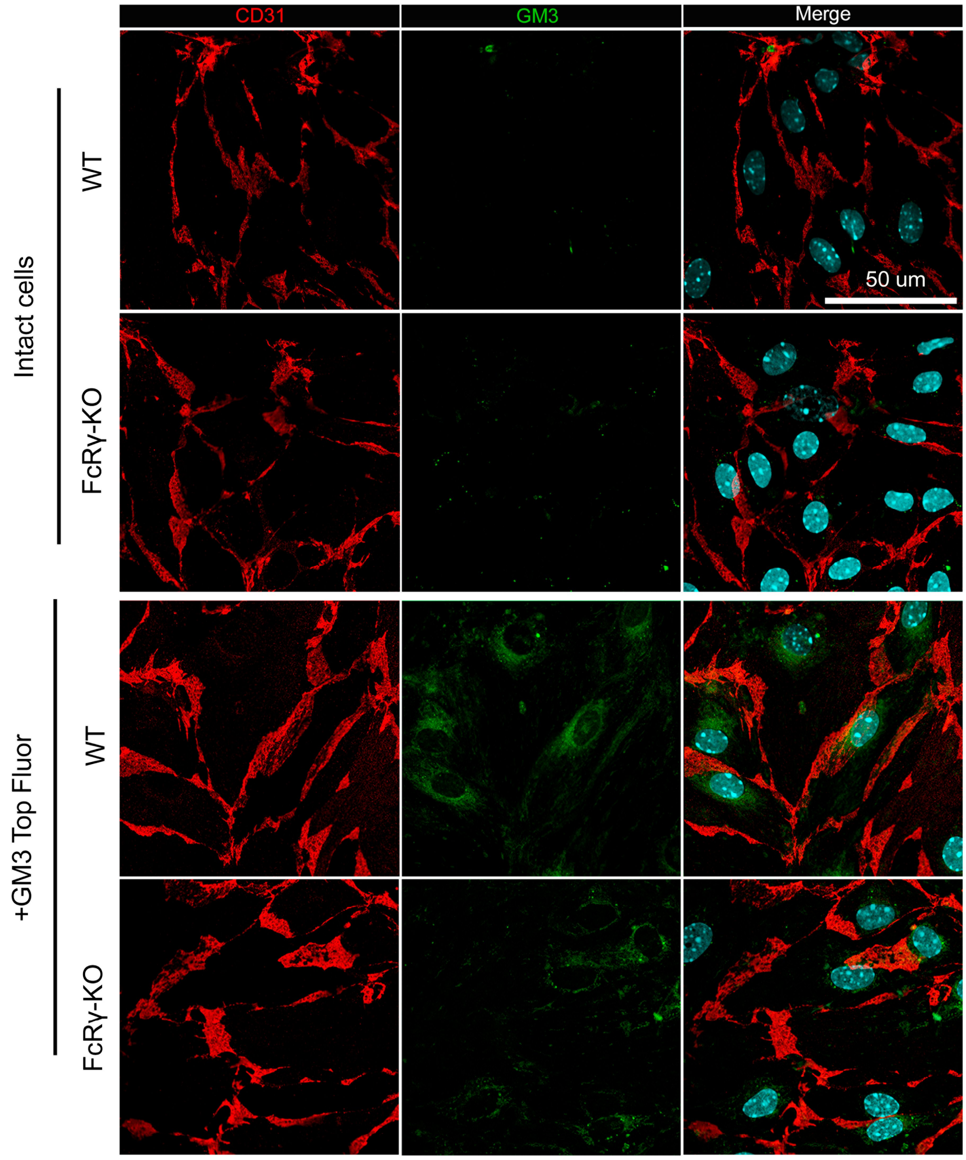

Supplementary Fig. 8. FcRγ-deficiency reduced uptake of GM3.

Brain endothelial cells isolated from FcRγ-KO mice and wild-type (WT) mice via puromycin selection were incubated with TopFluor-labelled ganglioside GM3 (green) for two hours, stained using anti-CD31 antibodies (red) to delineate cell boundaries and evaluated by confocal microscopy. Nuclei were stained with DAPI (cyan). Representative results from two independent experiments are shown in the figure.

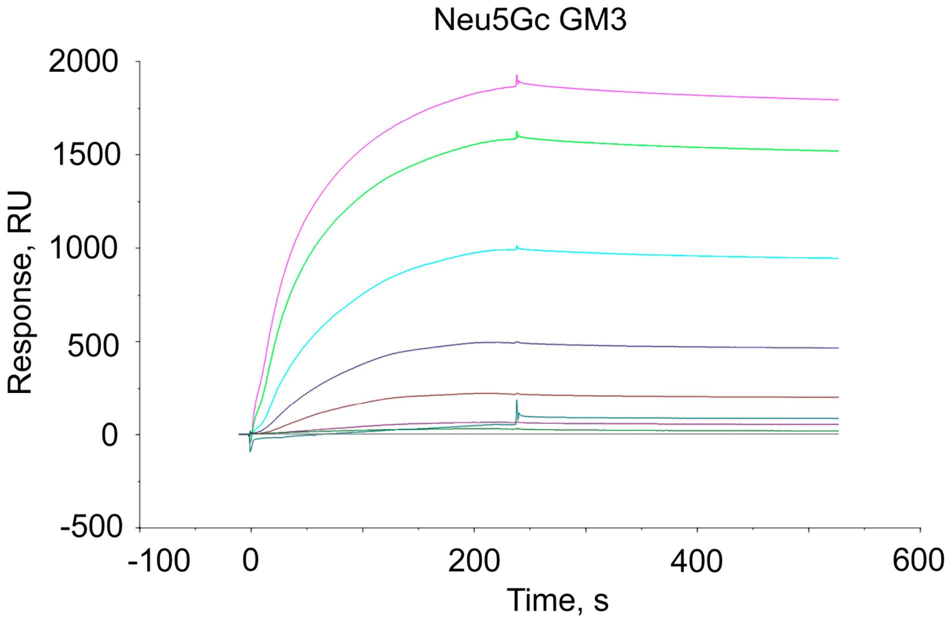

Supplementary Fig. 9. Mincle recognizes N-glycolyl-Ganglioside GM3 (Neu5Gc GM3) through direct binding.

SPR sensorgrams of Neu5Gc GM3 binding to chip-immobilized Mincle are expressed in RU vs. time after double referencing (blank surface and blank buffer referencing). Recombinant human Mincle was produced in human cells. The concentrations of Neu5Gc GM3 were 0.78, 1.56, 3.13, 6.25, 12.5, 25, 50 and 75 µM (from bottom to top). All data shown are representative of two independent experiments.

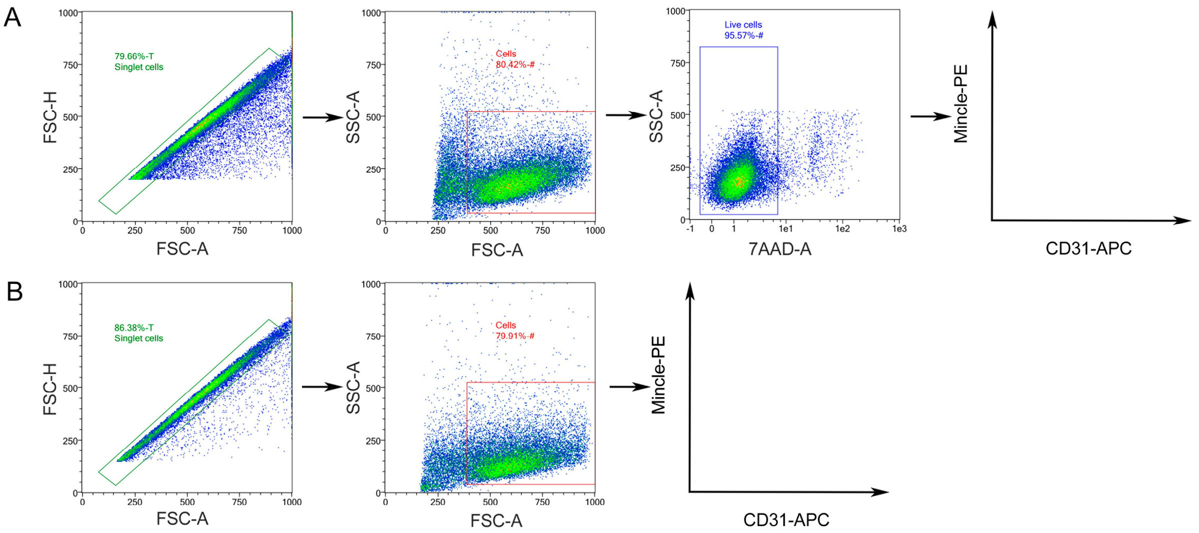

Supplementary Fig. 10. Gating strategy to assess Mincle and CD31 expression on HUVECs.

A. Gating strategy for evaluation of HUVECs without permeabilization. A forward scatter height (FSC-H) vs. forward scatter area (FSC-A) plot was used to exclude doublets or larger aggregates, and cells in this gate were further analysed with a SSC-A (side scatter area) vs. FSC-A dot plot to identify the original total cell population. The gated population was further analysed for the uptake of 7-aminoactinomycin D (7-AAD) to identify live cells. Surface expression of Mincle and CD31 was then evaluated in this gated population of live cells.

B. Gating strategy for evaluation of HUVECs with permeabilization. A FSC-H vs. FSC-A plot was used to exclude doublets or larger aggregates, and cells from this gated population were then analysed with a SSC-A vs. FSC-A dot plot to identify the original total cell population. Cells from the gated population were analysed for Mincle and CD31 expression.

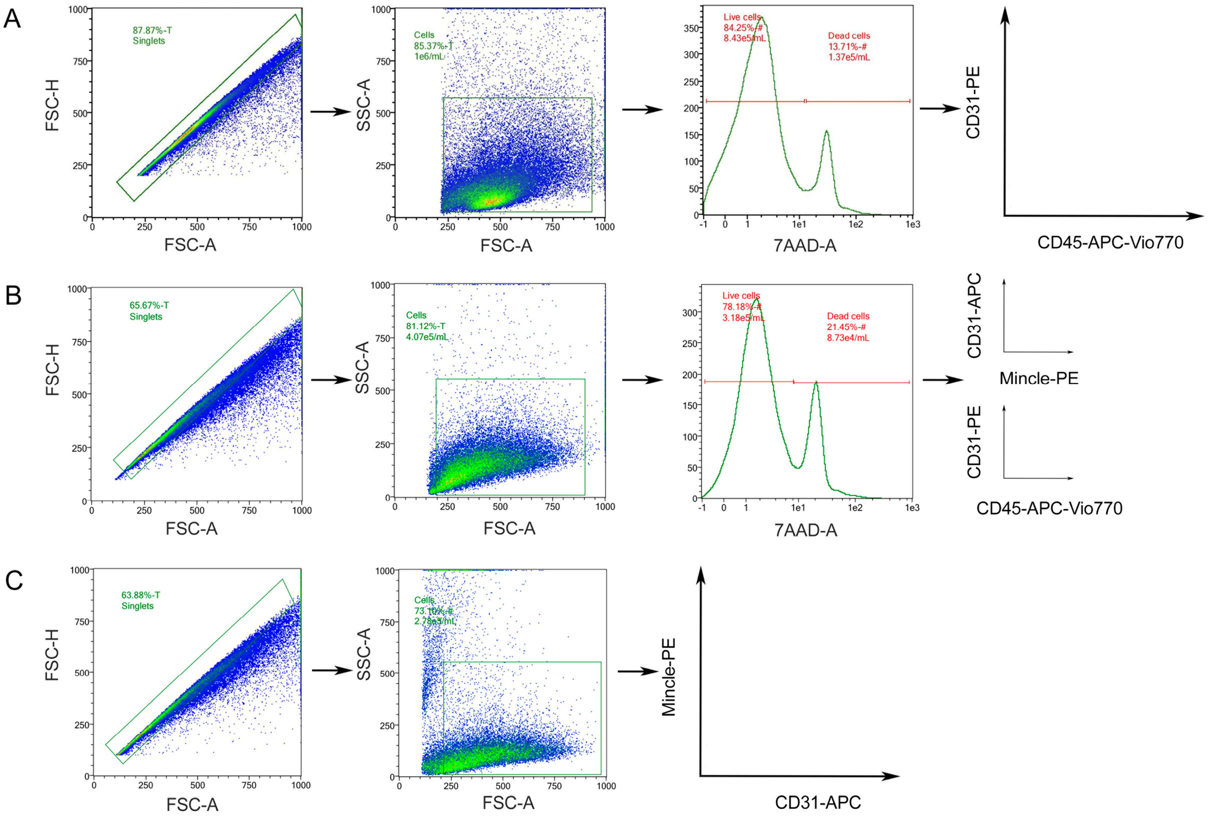

Supplementary Fig. 11. Gating strategy for flow cytometric analyses of mouse brain endothelial cells.

A. Flow cytometry was performed after enzymatic digestion to obtain a single-cell suspension from mouse brain tissue. A forward scatter height (FSC-H) vs. forward scatter area (FSC-A) plot was used to exclude doublets or larger aggregates, and cells in this gate were further analysed with a SSC-A (side scatter area) vs. FSC-A dot plot to identify the original total cell population. The gated population was further analysed for the uptake of 7-aminoactinomycin D (7-AAD) to identify live cells. Surface expression of CD31 and CD45 was then evaluated in this gated population of live cells.

B. Gating strategy for analyses of live endothelial cells isolated from mouse brains using MACS or puromycin selection. A FSC-H vs. FSC-A plot was used to exclude doublets or larger aggregates, and cells in this gate were further analysed with an SSC-A vs. FSC-A dot plot to identify the original total cell population. The gated population was further analysed for the uptake of 7-AAD to identify live cells. Subsequent analyses were performed with this gated population of live cells. Cells from the gated population were analysed for Mincle, CD31 or CD45 expression.

C. Gating strategy for analyses of permeabilized endothelial cells isolated from mouse brains. A FSC-H vs. FSC-A plot was used to exclude doublets or larger aggregates, and cells in this gate were further analysed with an SSC-A vs. FSC-A dot plot to identify the original total cell population. Cells from the gated

population were analysed for Mincle and CD31 expression.
